# Antibacterial mechanisms of phenyllactic acid against *Shewanella putrefaciens* by metabolomics and machine learning and its application in sea fish

**DOI:** 10.3389/fnut.2026.1852332

**Published:** 2026-06-29

**Authors:** Yunpo Huang, Jinyi Yang, Zhen Zhang, Zheng Zhan, Mina Huang, Chen Li

**Affiliations:** 1 College of Food and Health, Jinzhou Medical University, Jinzhou, China; 2 Liaoning Provincial Professional Technology Innovation Center of Meat Processing and Quality-Safety Control, Jinzhou, China; 3 School of Nursing, Jinzhou Medical University, Jinzhou, China

**Keywords:** antibacterial, machine learning, metabolomics, phenyllactic acid, *Shewanella putrefaciens*

## Abstract

*Shewanella* is a common spoilage bacterium found in aquatic products, and inhibiting its growth is currently a critical strategy in seafood preservation. In this study, a comprehensive analytical method combining metabolomics and machine learning was used to systematically elucidate the antibacterial mechanisms of phenyllactic acid (PLA) against *Shewanella putrefaciens* SP22. The minimum inhibitory concentration of PLA was 2.0 mg/mL. PLA disrupts the cell wall integrity, changes cell membrane permeability and integrity, and inhibits key enzyme activities. Non-targeted metabolomic analysis revealed 1,211 significant differential metabolites between PLA treatment and control groups, mainly involving the citric acid cycle, amino acid synthesis, nucleotide metabolism, glycerophospholipid metabolism, and cofactor generation metabolic pathways. Four machine learning models were constructed based on metabolomics data, and key biomarkers (such as citric acid, *α*-ketoglutaric acid, and 5′-adenylate nucleotide) were screened. Meanwhile, the antibacterial and preservative effects of PLA were verified in large yellow croaker, salmon, and sea bass. This study lays a theoretical foundation for applying PLA as a natural preservative for aquatic product preservation.

## 1 Introduction

The prevention and control of foodborne spoilage bacteria remain a key focus of current food safety research. According to statistics from the Food and Agriculture Organization of the United Nations (FAO), approximately 30–35% global aquatic products are lost annually due to spoilage. Owing to its exceptional cold tolerance and spoilage activity, *Shewanella* can maintain a high growth rate under refrigerated conditions (4 °C), making it a specific spoilage bacterium in aquatic products (1). It can compromise sensory quality by causing flesh degradation, slime formation, and H_2_S production, among other factors, resulting in major economic losses for the aquatic product and meat processing industries, particularly in seafood products such as yellow croaker (2). Furthermore, consuming seafood contaminated with *Shewanella* spp. can cause diseases such as gastroenteritis, otitis media, and cellulitis, which can pose a serious threat to public health. However, traditional chemical preservatives carry safety risks and can induce resistance, while physical treatment technologies often compromise aquatic product quality (1). Therefore, developing safe, efficient, and natural antimicrobial strategies to control the spoilage activity of *Shewanella putrefaciens* in aquatic products is of great significance.

Phenyllactic acid (PLA) is an organic acid naturally present in fermented foods and widely found in traditional fermented products such as yogurt, kimchi, and fermented meat products (3–5). As a naturally occurring antimicrobial substance derived from food matrices, PLA has garnered significant attention in food preservation owing to its broad-spectrum antimicrobial activity and excellent biosafety. It has been applied to preserve various foods, including bread, corn, and dairy products (4). Pius Bassey et al. (6) have demonstrated that treatment with PLA solutions significantly reduced the total bacterial count, total volatile basic nitrogen (TVB-N), and thiobarbituric acid reactive substances (TBARs) in fresh pork loins. However, PLA application in aquatic products has not been reported to date. Current research primarily focuses on the macroscopic antibacterial effects and phenotypic changes of PLA. Systematic analysis of the precise molecular mechanisms by which PLA interferes with *S. putrefaciens* metabolic networks and targets specific key metabolic pathways is still lacking. Therefore, elucidating the molecular mechanisms by which PLA acts on *S. putrefaciens* is of great significance for its development and application as a novel natural food preservative.

Machine learning (ML) is an analytical technique that automatically learns patterns from data to predict or discover potential correlations (7–9). Recently, this approach has demonstrated tremendous potential in food safety and quality monitoring. Cao et al. (10) combined metabolomics with ML to analyze changes in volatile metabolites during the refrigerated storage of goose eggs, successfully identifying four potential spoilage markers. Similarly, Mousavi et al. (11) combined electrochemical sensors with ML to develop a rapid and reliable method for detecting meat spoilage. These studies highlight the practicality of ML in food quality assessment, but primarily focus on spoilage detection or biomarker identification rather than elucidating mechanisms. To date, combining ML with physicochemical characterization and metabolomics to systematically investigate the antimicrobial mechanisms of small-molecule compounds remains largely unexplored. In this study, we utilized ML techniques to analyze high-dimensional metabolomic data, aiming to identify key metabolic biomarkers and further elucidate the multi-target antimicrobial mechanism of PLA against *S. putrefaciens*.

To address this research gap, this study proposes a comprehensive research strategy that integrates traditional microbiological analysis, metabolomics, and ML. First, we systematically characterized the effects of PLA on the cellular structure of *Shewanella* species using techniques such as liquid culture, scanning electron microscopy (SEM), Fourier-transform infrared spectroscopy (FTIR), and confocal laser scanning microscopy (CLSM). Second, we comprehensively analyzed the PLA treatment-induced changes in overall metabolic profile using non-targeted metabolomics techniques to elucidate its regulatory mechanisms at the molecular level; based on this, we introduced ML algorithms to screen for and predict key metabolic biomarkers. Finally, we preliminarily explored its application potential and economic value by evaluating the actual preservation effects of PLA on various aquatic fish species. In summary, this study aims to systematically elucidate the antimicrobial mechanism of PLA against spoilage-causing *Shewanella* bacteria and its preservation applications, from macro-phenotypic and molecular mechanisms to intelligent prediction systems, thereby providing a scientific basis for the development and application of PLA as a novel natural food preservative.

## 2 Materials and methods

### 2.1 Chemicals and bacterial strains

PLA and propidium iodide (PI) were purchased from Sigma Trading (Shanghai, China). *S. putrefaciens* SP22 was obtained from the Bohai University Laboratory (Jinzhou, China). The detection kits used in the experiment were purchased from Nanjing Jiancheng Reagent (Nanjing, China).

### 2.2 Antibacterial activity

#### 2.2.1 Minimum inhibitory concentration and minimum bactericidal concentration determination

The antibacterial activity and MIC of PLA against *S. putrefaciens* SP22 was determined using a combination of liquid culture and plate spreading methods. PLA mixed with 1 × 10^6^ CFU/mL at final concentrations of 0.5, 1, 2, and 4 mg/mL was incubated at 28 °C for 24 h. After incubation, the turbidity of the culture in the tubes was observed, and 100 μL culture was 10-fold serially diluted followed by colony counting. The MIC was the lowest PLA concentration that inhibited growth after 24 h of incubation at 28 °C. The MBC of PLA against *S. putrefaciens* SP22 was determined by subculturing the content of the wells with no visible growth on nutrient agar plates.

#### 2.2.2 Time-killing curve

Slight modifications were made to the experimental method described by Zhang et al. (12). PLA was added to the cell suspension at a concentration of 1 × 10^6^ CFU/mL to obtain final concentrations of 1/2 MIC, MIC, and 2 MIC. The cell suspensions were incubated at 28 °C for certain periods, the cells were inoculated into nutrient broth (NB) plates, and the colonies were counted after incubation at 28 °C for 2, 4, 6, 8, 12, and 24 h. Time-killing curves were plotted using the relationship between average colony count (log_10_ CFU/mL) and time.

#### 2.2.3 Growth curve and first-order kinetics fitting analysis

The growth curves of *S. putrefaciens* SP22 were fitted using the SGompertz model to construct a first-order mathematical model (13). This model is given by Equations 1–3:


y=A×exp(−exp(−μ(t−tc))) (1)

λ=tc−(1/μ) (2)

μmax=A×μ/e (3)

Where y is the OD_600_ of *S. putrefaciens* at time t; A is the OD_600_ at the maximum bacterial quantity (initial OD_600_ value is 0); μ is the relative growth rate at time tc, which is the time consumed at the maximum growth rate, (i.e., the inflection point of the growth curve); *λ* is the time until the lag period ends; and μmax is the maximum growth rate achieved. The goodness of fit was evaluated using the coefficient of determination *R*^2^.

### 2.3 Morphology

As described by Ning et al. (14), the surface morphology of *S. putrefaciens* SP22 cells in samples treated with PLA for 4 h was observed using SEM.

### 2.4 PI staining analysis

According to the method described by Zhong et al. (15), the blank control group was treated with an equal volume of phosphate-buffered saline (PBS), whereas the experimental groups were treated with 0.5 MIC, MIC, and 2 MIC PLA. The samples were observed using CLSM at an excitation wavelength of 546 nm.

### 2.5 Cell integrity and permeability

#### 2.5.1 Fourier-transform infrared spectroscopy

An equal volume of bacterial suspension was mixed with PLA thoroughly (final concentration considered as MIC) with PBS as the control, and incubated at 28 °C for 24 h. The samples were centrifuged at 4000 rpm for 10 min to collect the bacteria. An FTIR spectrometer (Thermo Fisher Nicolet IS50) was used to scan 32 times at a 400–4,000 cm^−1^ to determine the transmittance.

#### 2.5.2 Nucleic acid and protein leakage

Indicator bacteria were cultured in sodium chloride crystal violet enrichment medium until they reached the logarithmic growth phase (16). The cells were washed thrice with PBS and diluted to 10^8^ CFU/mL. The experimental groups were 1/2 MIC, MIC, and 2 MIC PLA, with the PBS buffer solution as the control group. They were incubated at 28 °C for 3 h. The OD at 260 and 280 nm were measured using a UV spectrophotometer. The results were averaged after three parallel measurements.

### 2.6 Key enzyme activity determination in energy metabolism

#### 2.6.1 Alkaline phosphatase, ATPase, and pyruvate kinase activities

According to the method described by Li et al. (16) with minor modifications, logarithmic phase *S. putrefaciens* SP22 cells were diluted in 0.85% NaCl solution to 2 × 10^8^ CFU/mL. These cells were mixed with PLA to form experimental groups at1/2 MIC, MIC, and 2 MIC. After incubation for 0, 3, 6, 9, and 12 h, the alkaline phosphatase (AKP) kit was used. The ATPase and pyruvate kinase (PK) contents were measured at 636 and 340 nm, respectively. Each sample was tested thrice in parallel.

#### 2.6.2 Isocitrate dehydrogenase, malate dehydrogenase, and succinate dehydrogenase activities

The mixed solution was treated as 2.6.1; the cells were sonicated at 200 W power in an ice water bath (for 3 s with a 7 s interval; total: 5 min) and centrifuged at 4 °C and 2,500 rpm for 10 min. The supernatant was used for testing. The intracellular isocitrate dehydrogenase (IDH), malate dehydrogenase (MDH), and succinate dehydrogenase (SDH) activities of the indicator bacteria were determined and calculated according to the manufacturer’s kit instructions at 450, 340, and 600 nm, respectively.

### 2.7 Nicotinamide adenine dinucleotide determination

The supernatant containing nicotinamide adenine dinucleotide (NAD^+^/NADH) was extracted as described by Chen et al. (17). The assay was performed using a commercial kit (Shanghai Sangon Biotech Co., Ltd., Shanghai, China).

### 2.8 Non-targeted metabolomics analysis

The cells were exposed to the MIC at 28 °C for 24 h (untreated cells were used as the control, and labeled as CK). The mobile phase consisted of 0.1% formic acid aqueous solution and acetonitrile (0.1% formic acid), and a gradient elution procedure was adopted on a Waters ACQUITY UPLC BEH C18 column (1.7 μm, 2.1 × 100 mm^2^). Mass spectrometry analysis was performed using a Thermo Q-Exactive Orbitrap with an electrospray ionization source in positive and negative ion modes, at a resolution of 70,000 (full scan), using data-dependent acquisition for MS/MS fragmentation. Data preprocessing was carried out using Compound Discoverer 3.3. Missing values were imputed using k-nearest neighbors (*k* = 3). Peak intensities were normalized by total ion current (TIC). Pooled quality control (QC) samples were injected every 10 runs, and features with >20% coefficient of variation (CV) in QC samples were excluded. Metabolite identification was performed at MSI level 2 using KEGG, HMDB, and METLIN databases. Statistical thresholds for differential metabolites were VIP > 1.0, |log2FC| > 1.5, and adjusted *p*-value (FDR < 0.05) using Benjamini–Hochberg correction (18). Detailed stepwise protocols are provided in Supplementary Methods S1.

### 2.9 ML analysis

For data mining and biomarker discovery, the analysis was framed as a binary classification task to identify metabolites that were significantly different between the PLA-treated (label:0) and control (label:1) groups (7–9). The processed dataset, comprising N samples and M metabolic features, was subjected to machine learning modeling. Four algorithms were used: RF, SVM, LR, and XGBoost. All model developments and evaluations were performed using Leave-One-Out Cross-Validation (LOOCV). Model performance was evaluated primarily based on the area under the receiver operating characteristic curve (AUC-ROC) and accuracy (ACC) aggregated across all LOOCV iterations. The SHapley Additive exPlanations (SHAP) framework was applied *post hoc* to interpret the models and identify key discriminatory metabolites. Data preprocessing for machine learning included log2 transformation, Pareto scaling, and k-NN imputation (*k* = 3) for missing values. The training dataset consisted of six parallel biological samples per group (6 PLA-treated, 6 control). Feature selection (VIP > 1.0 from OPLS-DA) was nested within each LOOCV iteration, applied only to the training fold to avoid data leakage. External validation was performed using an independent test set (*n* = 6, 3 per group). The raw dataset is provided in Supplementary Table S1.

### 2.10 Application of PLA in sea fish preservation

Fresh large yellow croaker, salmon, and sea bass were purchased from the local market in Jinzhou, China. The upper back muscles of the large yellow croaker and sea bass (5 × 1 × 1 cm^3^) and set them aside. The salmon was cut into 3 × 4 × 1 cm^3^ pieces and set aside as well. Soak The samples were soaked in PLA (at MIC) for 90 s, immediately placed in a refrigerated container at 4 °C for storage, and aerobically packaged in sterile polyethylene bags. The samples that were not soaked in PLA were used as the control group. On the 0, 2, 4, 6, and 8 day, the sample discoloration was recorded; ΔE, pH, and hardness were measured; according to GB 4789.2–2022, 5009.228–2016, and 5009.181–2016, TVC TVBN, and TBARS contents were measured, respectively (19). Sensory evaluation was performed using a 9-point hedonic scale (1 = dislike extremely, 9 = like extremely) to assess odor, texture, and overall acceptability of the fish samples at each storage time point. Detailed sensory scores are presented in Supplementary Table S2. Biological replicates: three independent experiments, each with three technical replicates. Storage conditions: 4 °C, aerobically packaged in sterile polyethylene bags. The initial microbial load of fresh fish samples was approximately 3.14–3.29 log CFU/g.

### 2.11 Statistical analysis

All experimental data were analyzed using the SPSS software (version 26.0; IBM Corporation, Armonk, NY, United States) and Origin 2026 (Origin Software Inc., La Jolla, CA, United States). Data are expressed as mean ± standard deviation of three independent experiments. For non-targeted metabolomics and machine learning, the training dataset consisted of six parallel biological samples per group (6 for PLA-treated and 6 for control). One-way analysis of variance followed by Tukey’s multiple comparison test was used to determine significant differences between groups. *p* < 0.05 was considered statistically significant.

## 3 Results and discussion

### 3.1 Antibacterial activity of PLA against *Shewanella putrefaciens* SP22

**Figure 1:**
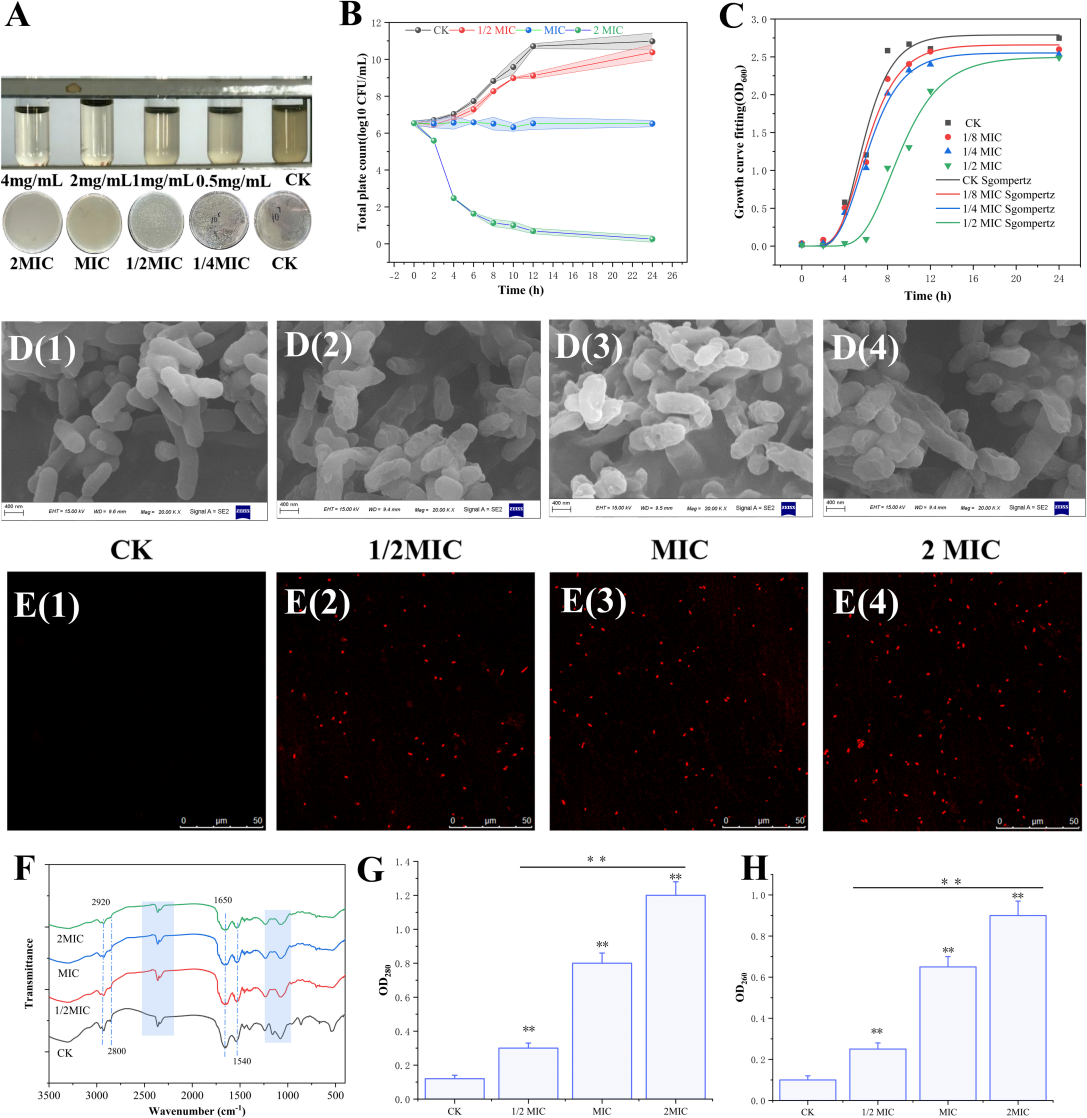
MIC Determination **(A)**; Growth curves of PLA-treated *S. putrefaciens SP22* with CK、1/2MIC, MIC, 2MIC **(B)**; The SGompertz model of PLA-treated *S. putrefaciens* SP22 with CK, 1/8MIC, 1/4MIC, 1/2MIC **(C)**; Morphological observation of *S. putrefaciens* SP22 under PLA treatment using scanning electron microscopy (SEM) **(D)**; Confocal laser scanning microscopy (CLSM) images of propidium iodide (PI)-stained *S. putrefaciens SP22* **(E)**. Effects of PLA (MIC) on cell wall and cell membrane integrity of *S. putrefaciens SP22.* FTIR analysis **(F)** DNA leakage **(G)**; Protein leakage **(H)**. * *p* < 0.05, ** *p* < 0.01, ***p < 0.001.

Distinct colonies were observed at 0.5 mg/mL and 1.0 mg/mL, while no colonies were observed at 2.0 mg/mL; furthermore, the bacterial solution was clear and transparent at 4.0 mg/mL (Figure 1A). The results indicate that the MIC of PLA is 2.0 mg/mL, lower than those previously reported by Ning et al. (3), Zhang et al. (12), and Chen et al. (20).

The CK group exhibited an S-shaped growth curve. As the PLA concentration increased, the bacterial count decreased exponentially, and the MIC ultimately decreased by three orders of magnitude, indicating that PLA possesses strong antimicrobial activity (Figure 1B).

Since bacterial growth was completely suppressed at MIC (2.0 mg/mL) and 2 MIC (4.0 mg/mL), only sub-MIC concentrations were used for growth curve analysis. The SGompertz model was used to fit the bacterial count curves. As shown in Figure 1C, The model parameters indicated that as the PLA concentration increased from CK to MIC, the maximum specific growth rate (*μ*) decreased from approximately 0.56 h^−1^ to approximately 0.38 h^−1^, and the lag phase (*λ*) extended from approximately 3.4 h to approximately 5.7 h (Table 1). The fitted curve closely matched the actual data points (*R*^2^ > 0.98), quantitatively confirming that PLA exerted antibacterial effects by prolonging the lag phase and reducing the growth rate.

**Table 1:** Kinetic parameters of *Shewanella putrefaciens* SP22 cells with different concentrations of PLA.

Concentration of PLA	A	Tc	μ	λ	μmax	*R* ^2^
CK	2.7896 ± 0.1544a	5.1545 ± 0.3257b	0.5644 ± 0.1395a	3.3825 ± 0.5458b	0.5792 ± 0.1467a	0.97858
1/8 MIC	2.6581 ± 0.1049ab	5.3089 ± 0.2351b	0.4925 ± 0.0788ab	3.2783 ± 0.4009b	0.4816 ± 0.0793b	0.99003
1/4 MIC	2.5518 ± 0.0811b	5.4491 ± 0.1886b	0.4811 ± 0.0602ab	3.3707 ± 0.3214b	0.4517 ± 0.0583bc	0.99387
1/2 MIC	2.4975 ± 0.1455b	8.2550 ± 0.3363a	0.3844 ± 0.0689b	5.6537 ± 0.5750a	0.3532 ± 0.0666c	0.98494

Different letters after the average of the same column indicate differences in statistics (*p* < 0.05).

### 3.2 Morphological analysis

Ultrastructural changes in *Shewanella* after PLA treatment were observed using SEM (Figure 1D). The CK group showed intact *S. putrefaciens* SP22 cells with a regular short rod shape, smooth surface, and clear contours. The PLA-treated group exhibited holes, deformation, wrinkles, fractures, and adhered deformed bacteria. No red-stained bacteria were detected in the CK group, indicating the absence of damaged cell membranes (Figure 1E). With increasing PLA concentration, the number of red-stained (PI-stained) bacteria gradually increased, suggesting that PLA damaged bacterial cell membrane integrity. A similar mechanism of bacterial inhibition has also been reported by Ning et al. (14), Deng et al. (21), and Li et al. (22, 23).

### 3.3 PLA-induced cell structural damage

#### 3.3.1 Fourier-transform infrared spectroscopy

The PLA-induced damage to *Shewanella* cell structure was analyzed through collective vibration changes of functional groups. The PLA-treated group exhibited a distinct blue shift in the –CH_2_ stretching vibration peaks within the 3,000–2,800 cm^−1^ region (at approximately 2,920 and 2,850 cm^−1^) than the CK group (Figure 1F) (24). The absorption peaks corresponding to the amide I band (*α*-helix, approximately 1,650 cm^−1^) and amide II band (approximately 1,540 cm^−1^) in the 1700–1,600 cm^−1^ region were significantly weakened with altered peak shapes (25). The intensity of absorption peaks within the 1,300–900 cm^−1^ region (at approximately 1,250 cm^−1^ and 1,080 cm^−1^) markedly decreased and became less distinct Marangon et al. (26). These spectral changes indicate that PLA disrupts membrane stability and permeability, induces protein secondary structure extensive denaturation or degradation, and leads to disordered arrangement and compromised integrity of membrane phospholipids. Additionally, alterations in polysaccharide-related peaks within the 1,200–900 cm^−1^ range suggest a concomitant impact on cell wall structure.

#### 3.3.2 Nucleic acid and protein leakage

The OD_260_ and OD_280_ values of the CK group were low (0.17 ± 0.02 and 0.18 ± 0.01, respectively), indicating intact cell membrane (Figures 1G,H). The OD_260_ and OD_280_ values of the MIC group were 0.65 ± 0.01 and 0.66 ± 0.01, respectively, which were approximately 3.8 and 3.7 times those of the CK group, respectively. Therefore, the increased absorbance of the supernatant directly proved that the cellular contents leaked out of the cell due to membrane damage and reduced cell membrane integrity. Zinc oxide nanoparticles (ZnO-NPs) against *Shewanella putrefaciens* indicated the same results (27).

### 3.4 Changes in energy metabolism levels

#### 3.4.1 AKP, ATPase, PK, IDH, SDH, and MDH activities

**Figure 2:**
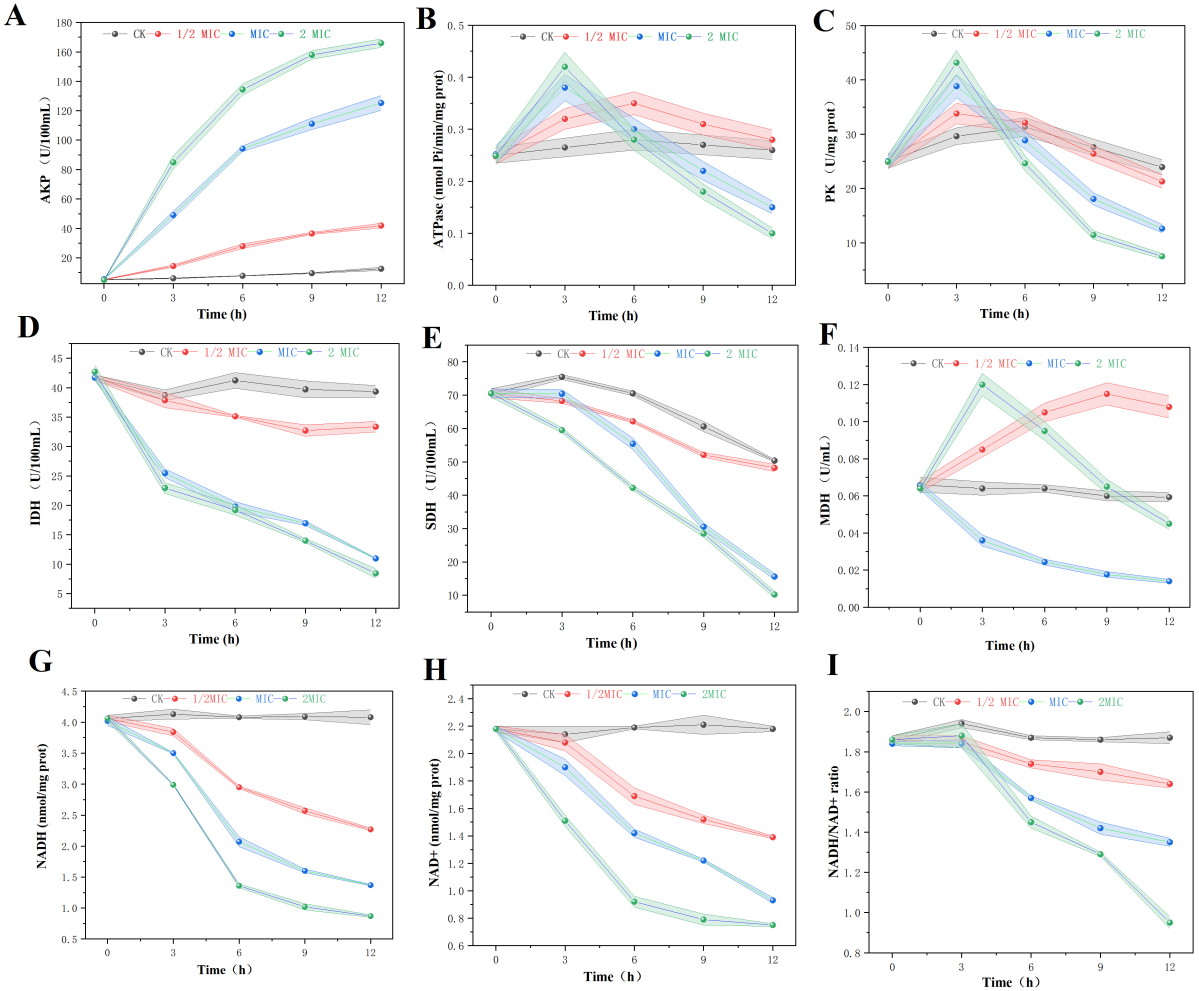
Effects of PLA (MIC) on intracellular substance activities in *S. putrefaciens* SP22. Alkaline phosphatase (AKP) activity **(A)**; Change in ATPase activity **(B)**; Change in pyruvate kinase (PK) activity**(C)**; Change in isocitrate dehydrogenase (IDH) activity**(D)**; Change in succinate dehydrogenase (SDH) activity **(E)**; Change in malate dehydrogenase (MDH) activity **(F)**; Change in NADH content **(G)**; Change in NAD^+^ content **(H)**; Change in the NADH/NAD^+^ratio **(I)**.

ATPase, PK, IDH, SDH, and MDH activities in the PLA treatment group was consistently significantly lower than that in the CK group (Figure 2; *p* < 0.05). The AKP activity in the 2 MIC group was the highest, reaching 84.88 ± 4.56 and 165.96 ± 2.84 U/100 mL at 3 and 12 h, respectively, which was approximately 13.17 times that of CK group at the same time point (Figure 2A). Noticeably, all treatment groups showed an activity peak at 3 h, which increased with increasing concentration (Figures 2B,C). This “first increase then decrease” change in ATPase and PK activities might be due to the compensatory stress response of bacteria to membrane damage (16). As PLA concentration increased, IDH activity decreased over time (Figure 2D). After MIC exposure for 12 h, the enzyme activity decreased to 10.97 ± 0.06 U/mL, which was 73.69% lower than that of the CK group. The SDH activity in the MIC group was close to that in the CK group from 0 to 3 h, and dropped sharply to 15.59 ± 0.44 U/mL at 12 h (Figure 2E). Figure 2F shows that the effect of PLA on MDH activity exhibited significant dose and time-dependent kinetic characteristic. The PLA treatment group exhibited sustained inhibition of enzyme activity (*p* < 0.05), indicating that PLA disrupts the energy metabolism of the indicator bacteria, thereby reducing energy production and inhibiting normal microbial growth. This finding is consistent with a previous report (28).

#### 3.4.2 NAD(H) cofactor quantification and NADH/NAD^+^ ratio

PLA treatment continuously and synchronously decreased intracellular NAD^+^ and NADH levels within 0–12 h, and significantly decreased the NADH/NAD^+^ ratio (Figures 2G–I). This abnormal phenomenon indicates that PLA treatment acts as a biological stress, severely inhibiting NAD synthesis pathway and continuously decreasing its total amount (29). The deficiency of NADH, the main substrate of the electron transport chain, hinders oxidative phosphorylation, sharply reduces ATP production, and leads to insufficient energy synthesis. This may be one of the core metabolic mechanisms by which PLA inhibits bacterial growth.

### 3.5 Metabolomics

#### 3.5.1 Screening and identification of differential metabolites

**Figure 3:**
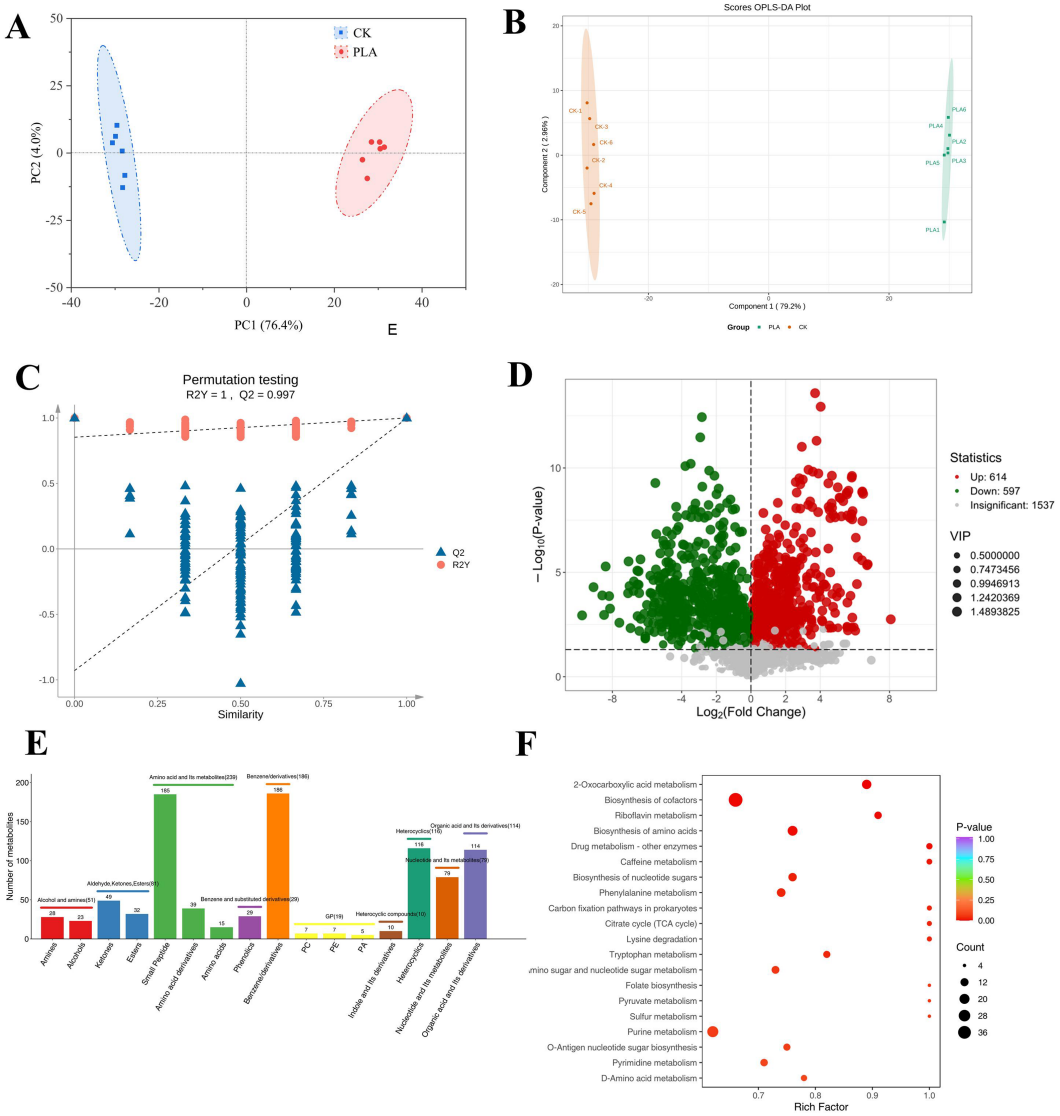
Untargeted metabolomics analysis of differential metabolites in *S. putrefaciens* SP22 after PLA (MIC) treatment. Principal component analysis (2D PCA plot) of samples in PLA vs. CK **(A)**; Orthogonal partial least squares discriminant analysis (OPLS-DA) of PLA vs. CK **(B)**; Permutation testing of OPLS-DA model **(C)** Bubble plot depicting differential metabolites in PLA vs. CK **(D)**; Bar chart showing the classification of differential metabolites in PLA vs. CK **(E)**; KEGG enrichment pathway map for PLA vs. CK **(F)**.

PCA indicated significant intergroup differences between the PLA and CK groups, and no dominant component within the groups (Figures 3A,B). PC1 explained 76.4% eigenvalue of the original dataset, and the metabolic changes in the experimental and control groups were significant. Supervised analysis using OPLS-DA effectively eliminated irrelevant influences not related to this study, with no overlap. The testing result of the Q2 was 0.997, R2Y = 1 (Figure 3C), indicating that the established OPLS-DA model could be used for the identification. Figure 3D shows that 2,847 metabolites were identified, of which 1,211 were significantly different, 614 were upregulated, and 597 were downregulated. Figure 3E shows the differential metabolites such as amino acids and their metabolites (239), benzene and its substituted derivatives (186), heterocyclic compounds (116), and others.

KEGG pathway enrichment analysis revealed the relationships between the differential metabolites (Figure 3F), highlighting a consistent downward trend across the top 20 key metabolic pathways. The key pathways through which PLA influences *Shewanella* metabolism were identified, including energy metabolism (TCA cycle), amino acid metabolism, nucleotide metabolism, glycerophospholipid metabolism, and the biosynthesis of cofactors. Li et al. (16) also reported similar results, finding that CO_2_ inhibited *S. putrefaciens* proliferation by interfering with energy, amino acid, respiratory chain, lipid, and nucleic acid metabolisms. Therefore, exploring the antibacterial effects of PLA at the level of metabolic pathway regulation in indicator bacteria is necessary.

#### 3.5.2 Analysis of the metabolism pathways

##### 3.5.2.1 Citric acid cycle

**Figure 4:**
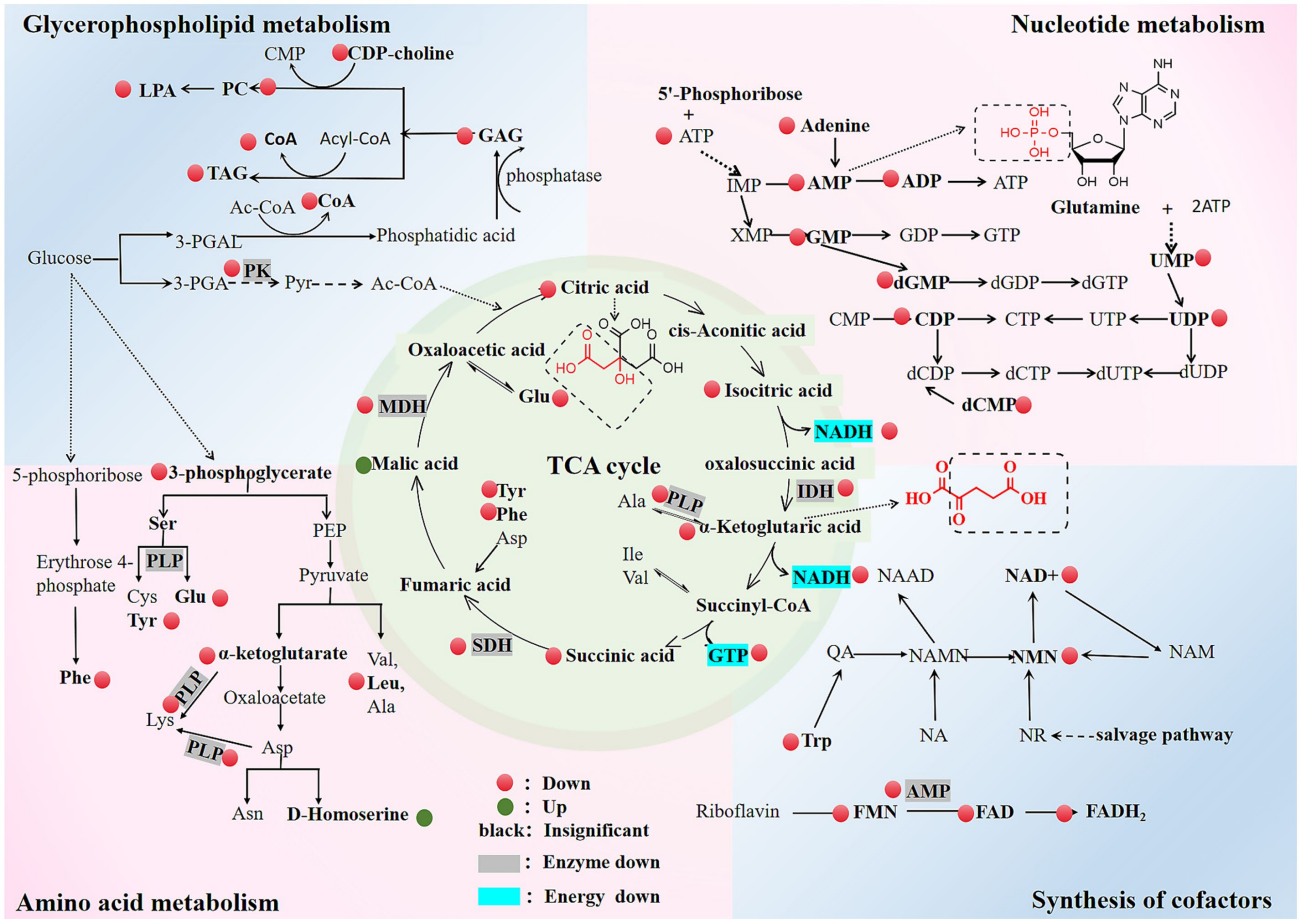
Metabolic pathway analysis of *S. putrefaciens* SP22. Red indicates significantly downregulated metabolites; green indicates significantly upregulated metabolites; black indicates metabolites with no significant difference.

The TCA cycle is the core mechanism by which microorganisms obtain large amounts of free energy (18, 30, 31). PLA significantly downregulates key intermediates (citric acid, succinic acid, *α*-ketoglutaric acid, and isocitric acid), while slightly upregulating malate, indicating disrupted energy metabolism (Supplementary Table S3; Figure 4). PLA limits acetyl-CoA availability, impairing citric acid synthesis and its downstream product isocitric acid. PLA further blocks isocitrate conversion to *α*-ketoglutarate and inhibits α-ketoglutarate conversion to succinate by reducing cofactors (CoA, FAD, NAD^+^) (32, 33). Additionally, PLA suppresses MDH activity, preventing malate-to-oxaloacetate conversion and causing malate accumulation. In summary, PLA systematically blocks the energy metabolism hub by synergistically inhibiting key enzymes and cofactors of the TCA cycle through multiple targets. This may be one of the core mechanisms underlying its highly effective antibacterial activity.

##### 3.5.2.2 Amino acid metabolism

Amino acids serve as not only the fundamental building blocks of proteins, but also essential precursors for key nitrogen-containing compounds, including amines, glutathione, nucleotides, and their coenzymes (23). Under PLA treatment, significant perturbations in amino acid metabolism were observed (Supplementary Table S3; Figure 4), with four significantly upregulated metabolites and 15 significantly downregulated metabolites. Notably, PLA markedly reduced the level of α-ketoglutarate, a critical intermediate in both the TCA cycle and nitrogen assimilation. This reduction impairs glutamate and glutamine synthesis, thereby disrupting protein biosynthesis, while also interfering with deamination and transamination reactions essential for nitrogen homeostasis (16, 17, 32).

Additionally, PLA treatment compromised cell wall integrity, triggering a cellular stress response that activates the peptidoglycan synthesis pathway. However, the availability of L-glutamate, a key substrate for this pathway, was significantly reduced, limiting peptidoglycan biosynthesis. Concurrently, PLA disrupted serine conversion to diaminopimelate (DAP), an essential component of peptidoglycan, further compromising cell wall repair. Collectively, these PLA-induced metabolic disturbances create a synergistic blockade that impairs both amino acid homeostasis and cell wall integrity, ultimately contributing to bacterial growth inhibition.

##### 3.5.2.3 Nucleotide metabolism

Purines and pyrimidines are essential components of nucleotide synthesis and their metabolism is closely related to energy metabolism and protein synthesis (16, 34). As shown in Supplementary Table S3 and Figure 4, four metabolites were upregulated and 14 were downregulated. Since CDP and UDP are key intermediates in CTP formation pathways, their downregulation indicates CTP synthesis inhibition. AMP and GMP are key intermediates in ATP formation catalysis, and their downregulation indicates ATP synthesis inhibition (35). In summary, nucleotide metabolites (such as AMP, ADP, UDP) and energy metabolites (such as NADP^+^, FAD) as well as TCA intermediates were significantly downregulated. This indicates that bacterial genetic material synthesis and the ATP generation pathway were simultaneously inhibited.

##### 3.5.2.4 Glycerophospholipid metabolism

**Figure 5:**
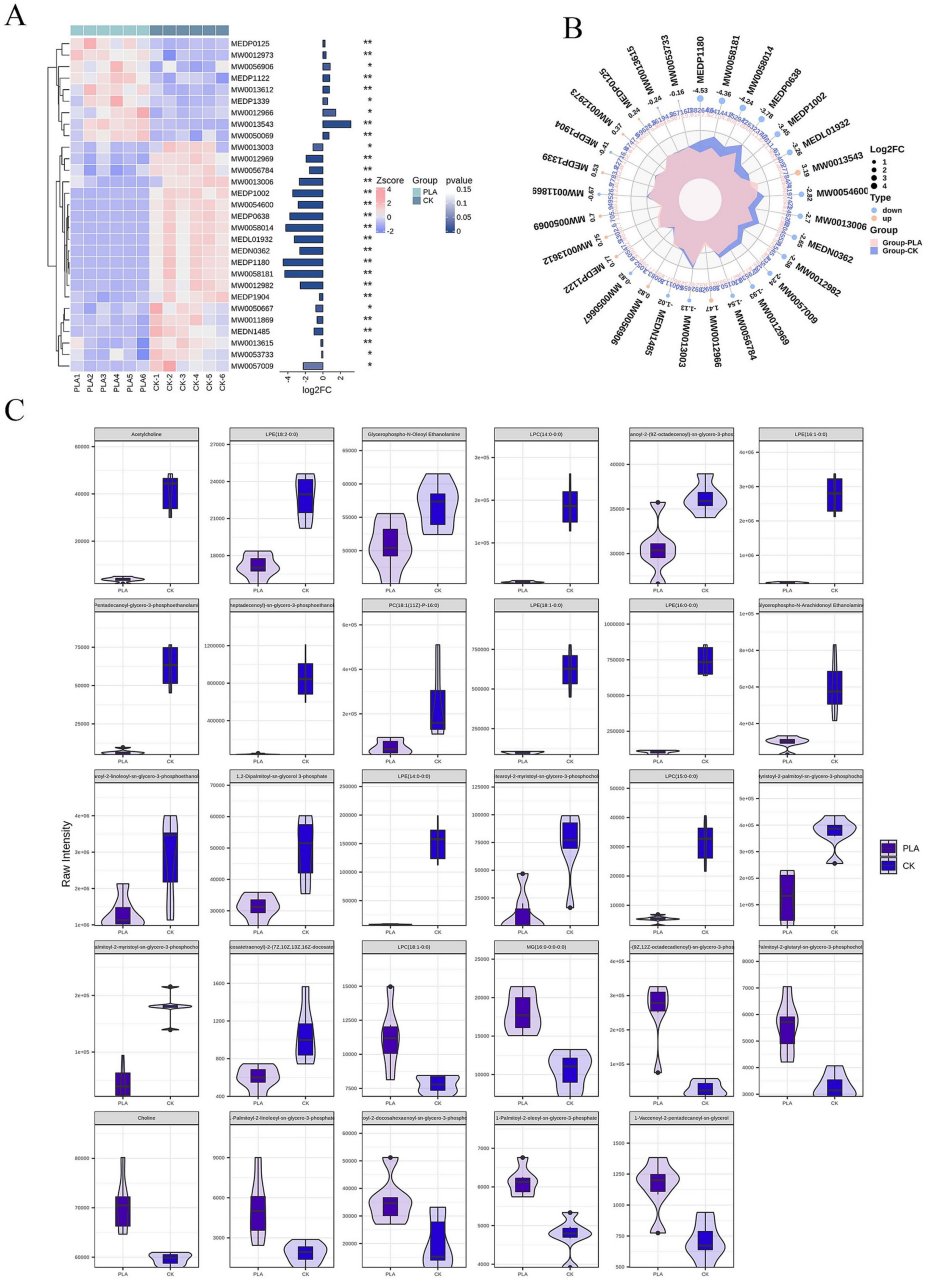
Heatmap of differential metabolites in glycerophospholipid metabolism (*p* < 0.05), with up-regulated metabolites in red and down-regulated metabolites in green **(A)**; Radar plot showing differential metabolites in glycerophospholipid metabolism in the PLA group compared to the CK group **(B)**. The sphere size is proportional to the absolute Log₂FC value, indicating the magnitude of the response; Box plot of differentially significant metabolites in the glycerophospholipid metabolic pathway **(C)**.

Among the 86 substances related to the glycerophospholipid metabolic pathway, 29 showed significant changes (9 were upregulated and 20 were downregulated; Figure 5A). Phospholipids are the main amphiphilic components of bacterial cell membranes. The hydrophilic regions of amphiphilic molecules are mostly simple –OH groups, which can easily form electrostatic binding or esterification interactions with the –COOH or phenolic groups of PLA under certain conditions. In addition, the hydrophobicity of PLA facilitates its insertion into the hydrophobic regions of PLs (36). This potential mechanism is consistent with physicochemical analysis showing that PLA disrupts cell membrane fluidity and integrity.

The metabolomics results in Figures 5B,C showed that phosphatidylethanolamine, and serine were significantly downregulated, indicating that the hydrophilic or charged substituents at the –C3 position of various phosphoglycerides decreased, and the core skeleton of phosphoglycerides, glycerol-3-phosphate, was downregulated, showing a trend of inhibitory regulation of glycerophospholipid synthesis metabolism. LPE(18:2/0:0) and LPE(14,0/0:0) are key products of membrane breakdown and were significantly downregulated, indicating that PLA interferes with membrane degradation. Notably, PLA, which are weakly ionized acidic small molecules, are prone to adsorption at the membrane site, causing membrane potential disorder and thus disrupting the metabolic function of the membrane (37, 38).

##### 3.5.2.5 Cofactor synthesis

Cofactors were the most significantly affected metabolite group under PLA treatment, with 34 downregulated and 9 upregulated cofactors across pathways including NAD(P)^+^ biosynthesis, TCA cycle, and nucleotide metabolism (Supplementary Table S3; Figure 4). PLA markedly downregulated NAD(P)^+^ biosynthesis intermediates, leading to NADPH depletion. This impaired glutathione regeneration, compromising the antioxidant defense system, and hindered fatty acid and nucleotide synthesis. By broadly suppressing the cofactors involved in redox reactions and energy metabolism, PLA inhibited DNA/RNA synthesis, energy production, and oxidative stress repair. As a weak acid, PLA likely disrupts the intracellular H^+^ environment and interferes with proton transfer in the electron transport chain, triggering cascading effects on multiple metabolic pathways. These findings suggest that the proton transfer chain may be a potential key target of PLA in *Shewanella*.

##### 3.5.2.6 Potential mechanism

**Figure 6:**
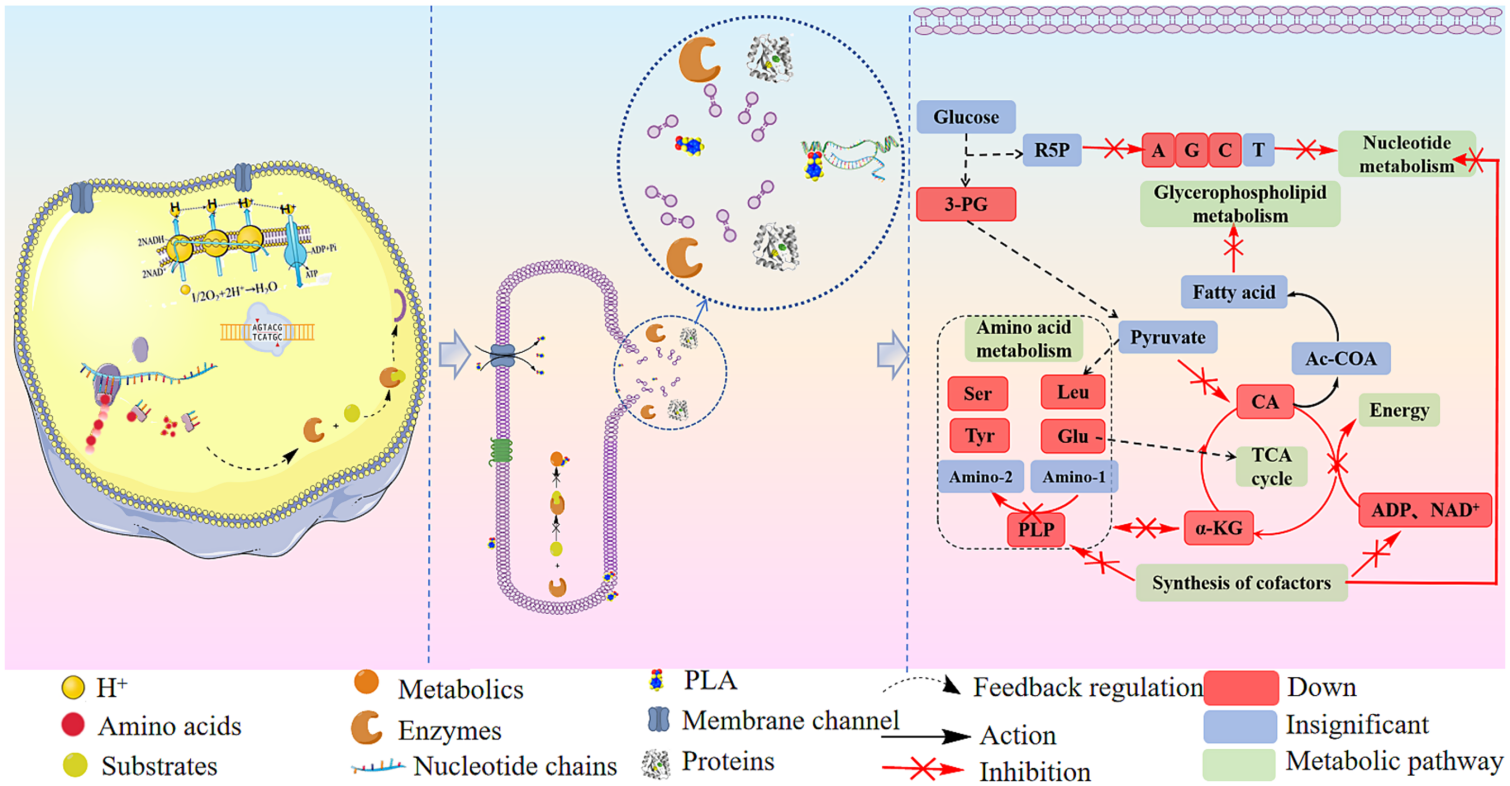
Antibacterial mode of PLA against *S. putrefaciens* SP22.

Our research was based on previous phenotypic data, combined with the results of non-targeted metabolomics and KEGG pathway enrichment analysis, to propose a structural–molecular mechanism model for *S. putrefaciens* SP22 inhibition by PLA. Specifically, PLA (at MIC) irreversibly damaged the rigid cell wall structure and cell membrane integrity and permeability, causing enzyme leakage and cellular content outflow (Figure 6). Simultaneously, PLA enters the bacterial cytoplasm through membrane channels or membrane pores and regulates metabolic pathways during bacterial stress resistance. The main manifestation is that the key metabolites of key pathways are inhibited, metabolic intermediate products undergo material transformation and lose their intermediate function, and the proton transfer chain is markedly disrupted. The inhibitory effect of PLA is not a single inhibition or activation, but a cascade of metabolic regulation through multiple targets and pathways, ultimately resulting in blocked material transformation, insufficient energy supply, and metabolic imbalance, achieving an antibacterial effect. Specifically, the decreased IDH, SDH, and MDH activities (Figure 2) were directly associated with reduced isocitrate and *α*-ketoglutarate abundances and malate accumulation (Supplementary Table S3; Figure 4), indicating a causal cascade from enzyme inhibition to metabolic blockade.

##### 3.5.2.7 Role of pH vs. specific activity

The pH of the PLA solution at MIC (2.0 mg/mL) was 3.8, while that of the control was 6.8. Acidic conditions can inhibit bacterial growth by disrupting membrane gradients. However, the observed multi-pathway metabolic disruptions (such as nucleotide and cofactor metabolism) are not typical of acid stress alone, suggesting that PLA exerts specific molecular actions. Future experiments with pH-matched controls (such as using HCl) are required to quantitatively separate pH-dependent effects from specific antibacterial activity of PLA.

### 3.6 Metabolic marker screening based on ML models

#### 3.6.1 Effectively distinguishing *Shewanella* strains treated with PLA

All models had excellent discriminative capabilities (AUC = 1.0000 for each model; Tables 2, 3). The RF and LR models achieved perfect classification (with ACC and F1 values of 1.0000), whereas SVM and XGBoost demonstrated extremely high efficiency (ACC = 0.9167, F1 value = 0.9231). The result of an AUC value of 1.0000 confirmed, from a computational perspective, an absolutely separable and clear systematic difference between the metabolic profiles processed by the PLA-treated and control groups, providing solid data guarantee for further in-depth exploration of its biological basis. To further assess generalizability, an independent external test set (*n* = 6, 3 per group) was used for validation, yielding an average AUC of 0.944 and ACC of 0.889, confirming that the key biomarkers remain robust despite the perfect AUC observed in LOOCV.

**Table 2:** Performance parameters of different machine learning models.

Model	AUC (LOOCV)	ACC (LOOCV)	F1_score (LOOCV)	Pre_score (LOOCV)	Recall_score (LOOCV)
RandomForest	1.0000	1.0000	1.0000	1.0000	1.0000
SVM	1.0000	0.9167	0.9231	0.8571	1
Logistic regression	1.0000	1.0000	1.0000	1.0000	1.0000
XGBoost	1.0000	0.9167	0.9231	0.8571	1.0000

**Table 3:** Hyperparameter settings of machine learning models.

Algorithm	Hyperparameters	Value
Xgboost	objective	binary:logistic
	eval_metric	logloss
	learning_rate	0.1
	n_estimators	100
	use_label_encoder	FALSE
RF	bootstrap	TRUE
	class_weight	balanced
	criterion	gini
	min_samples_leaf	1
	min_samples_split	2
	n_estimators	100
	max_features	sqrt
SVM	C	1
	cache_size	200
	decision_function_shape	ovr
	degree	3
	kernel	linear
	tol	0.001
Logistic regression	C	1
	fit_intercept	TRUE
	intercept_scaling	1
	max_iter	100
	penalty	l1
	solver	Liblinear
	tol	0.0001
	multi_class	deprecated

#### 3.6.2 Identification of key metabolic biomarkers based on SHAP analysis

**Figure 7:**
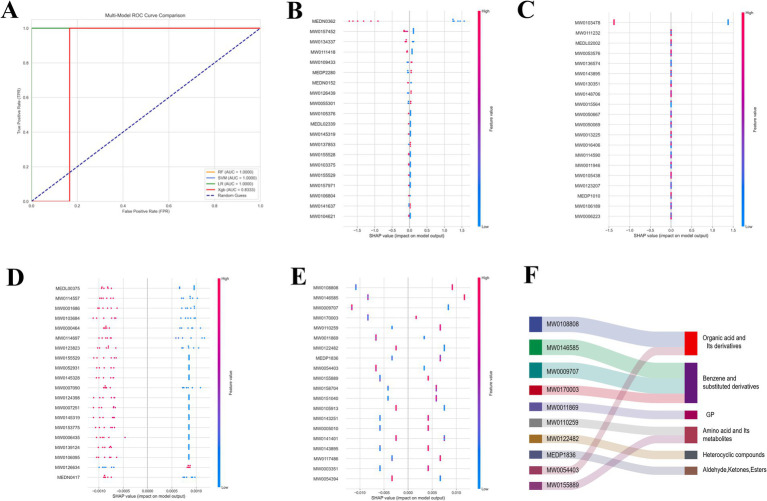
Comparison of ROC curves for different models **(A)**; SHAP plots for the RF **(B)**, LR **(C)**, XGB **(D)**, and SVM **(E)** models; The top 10 significantly different metabolite classifications **(F)** identified by the RF model screening.

Figure 7A shows that the performance of the four ML models is stable and reliable, and can be used for further analysis of metabolomics data. The RF model revealed complex interactions between the features (39). Key metabolites were successfully identified and covered six major categories: organic acids and their derivatives, benzene and its derivatives, glycerophospholipids, amino acids and their metabolites, heterocyclic compounds, aldehydes, ketones, and esters (Figure 7F). The characteristic disturbances in these metabolites in multiple dimensions revealed the potential core mechanism of the antibacterial effect of PLA. The metabolite ranked first in importance is MW0108808 (succinylated triethanolamine), which was positively correlated with the CK group classification (Figure 7B; Table 4). This means that a high content of this metabolite drove the samples toward the CK group classification. The key metabolite MW0009707 (salicin) showed a negative correlation. The higher the PLA content, the more likely is the model to classify the samples into the PLA group. Notably, the metabolite MW0170003 (veratramine) was not a common component of the classical bacterial metabolic network. This significant change may indicate that PLA treatment severely disrupts the membrane permeability or the external substance transport system of the bacteria. Although the exact role of this substance is unclear, the presence of veratramine provides new insights into the broad-spectrum antibacterial activity of PLA as a biomarker with high contribution value.

**Table 4:** The random forest model explains the top10 differential metabolites.

Index	Compounds	Class I	Class II	Rank
MW0108808	Nitrilotriacetic acid	Organic acid and Its derivatives	Organic acid and its derivatives	0.01
MW0146585	2,3-Benzofluorene	Benzene and substituted derivatives	Benzene and substituted derivatives	0.01
MW0009707	Salicin	Benzene and substituted derivatives	Benzene and substituted derivatives	0.01
MW0170003	Veratramine	Benzene and substituted derivatives	Benzene and substituted derivatives	0.006111
MW0011869	1,2-Dipalmitoyl-sn-glycerol 3-phosphate	GP	PA	0.005556
MW0110259	Tyr-Ile	Amino acid and Its metabolites	Small Peptide	0.005556
MW0122482	1-deoxy-1-(7-hydroxy-6-methyl-2,4-dioxo-3,4-dihydropteridin-8(2H)-yl)-D-ribitol	Heterocyclic compounds	Heterocyclic compounds	0.005417
MEDP1836	Nootkatone	Aldehyde, Ketones, Esters	Ketones	0.005278
MW0054403	Loganic acid	Organic acid and Its derivatives	Organic acid and Its derivatives	0.005278
MW0155889	Pro-Ile-Ala	Amino acid and Its metabolites	Small Peptide	0.005139

The analysis of the XGBoost model further confirmed these findings. The model identified metabolite MW0103478 (5’-adenylate nucleotide, AMP) as the most important feature. SHAP analysis clearly showed that low AMP content was a strong predictor for the PLA treatment group, whereas high AMP content was observed in the CK group (Figure 7C). The XGBoost model prioritizes the identification of AMP as a key biomarker, and this screening result is consistent with the core biomarkers determined in the nucleotide metabolic pathway analysis described in Section 3.5.2.4, forming a cross-validation at the ML and metabolic pathway levels.

The LR model, as a representative linearly interpretable model, shows that the metabolite MEDN0362 [lysophosphatidylethanolamine, LPE(18:1/0:0)] has a dominant influence. The absolute value of the SHAP score of this metabolite was much higher than that of all other features (Figure 7D), indicating that the model’s classification decisions were almost entirely driven by it. Its mode of action was clear and highly significant (*p* < 0.0001; Supplementary Table S3). Low levels of LPE (18:1/0:0) strongly drove the samples to be predicted as the PLA treatment group, whereas high levels led to the samples being classified as CK. This finding establishes specific perturbations in cell membrane phospholipid metabolism as the strongest linear discriminant factor for distinguishing between the treated and untreated states.

The SVM model provided another perspective for validation. The features with the highest contributions to this model included MW0103684 (UDP-glucose), MW0145328 (Arg-Gln-Leu), and MW0001686 (1-phenyl-1,2-propanedione). All showed a stable negative correlation with PLA group classification (Figure 7E). Such changes in these substances may directly disrupt the basic life activities of the bacteria. UDP-glucose is a direct precursor of bacterial cell-wall polysaccharides and glycogen (7, 9). A significant decrease in its content indicates that PLA inhibits bacterial cell wall synthesis and repair, as well as the energy storage process, thereby preventing the reversal of cell damage. Arg-Gln-Leu is a tripeptide, and the change in its level is a key indicator of the protein metabolic state. This significant decrease may reflect overall protein synthesis blockade, indicating that PLA interferes with bacterial amino acid metabolism and protein homeostasis.

### 3.7 Preservation effects on three sea fish pieces

#### 3.7.1 Appearance and color difference

**Figure 8:**
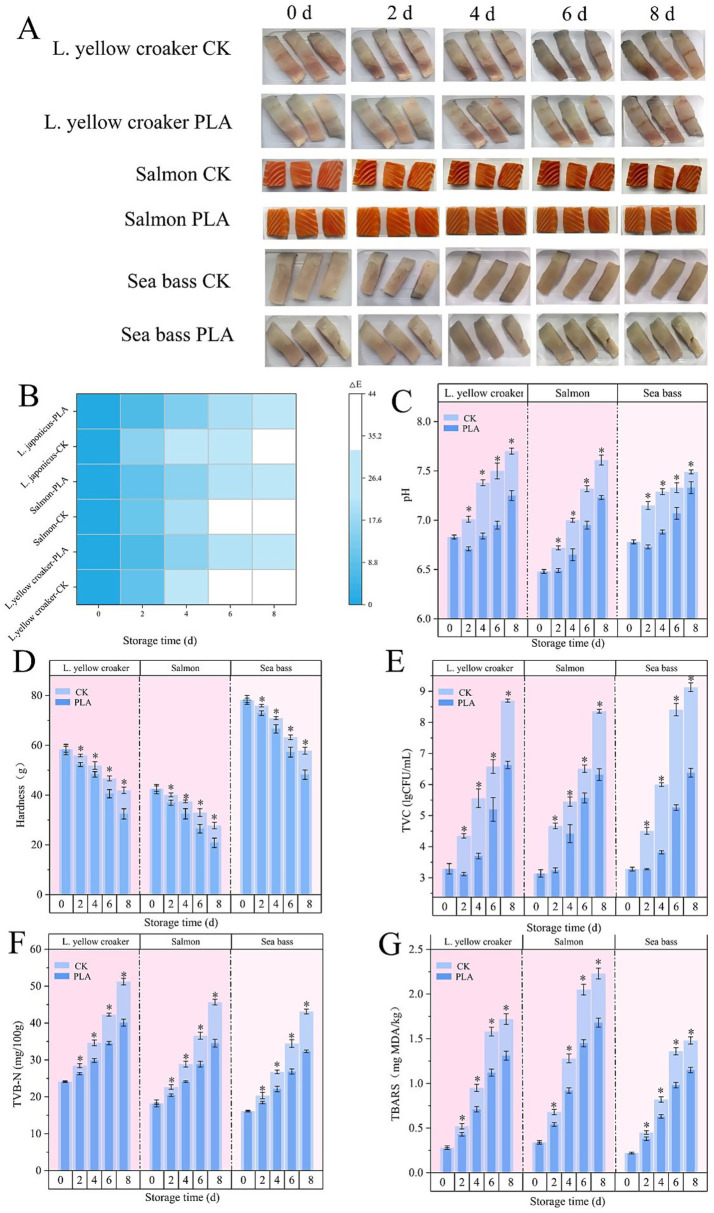
The effects of PLA treatment on the appearance **(A)**, color difference **(B)**, pH **(C)**, hardness **(D)**, TVC **(E)**, TVB-N **(F)**, and TBARS **(G)** of three types of sea fish. *p < 0.05, **p < 0.01, ***p < 0.001.

Figure 8A showed the changes in the appearance of the fish fillets. PLA had a good preservation effect on all samples. During the entire storage period, the morphology of the treated groups remained almost unchanged from the morphology of the fish at 0 days, showing an initial color, firm meat texture, and plump shape; in contrast, by 8 days, the fish fillets in the CK group showed severe deterioration, including increased discoloration, volume shrinkage, and obvious liquid leakage. The color difference also showed similar results (Figure 8B), PLA inhibited the color change of the fish pieces.

#### 3.7.2 Effects of PLA on the quality of three fish species

PLA treatment delayed quality deterioration in large yellow croaker, salmon, and sea bass during storage. PLA slowed the pH increase caused by alkaline metabolite accumulation (Figure 8C). Hardness declined in all groups (Figure 8D), but PLA significantly delayed muscle softening. By day 8, PLA reduced hardness loss from 44.34 to 28.20% in yellow croaker, from 50.88 to 24.40% in salmon, and from 38.42 to 26.34% in sea bass, with the strongest preservation in sea bass.

PLA also inhibited microbial growth (Figure 8E). At day 8, the TVC in PLA-treated samples was 6.63, 6.32, and 6.38 lg CFU/g for yellow croaker, salmon, and sea bass, respectively, representing 23.79, 24.40, and 30.12% reductions, respectively, compared to controls. Similarly, PLA delayed protein degradation and lipid oxidation (Figures 8F,G). The TVB-N values at day 8 were reduced by 21.70, 24.35, and 25.01%, while TBARS values were reduced by 23.84, 24.66, and 22.30% in the three species, respectively.

These results demonstrate that PLA effectively maintains fish quality by inhibiting microbial growth, delaying protein decomposition, and suppressing lipid oxidation, with species-specific efficacy (40).

#### 3.7.3 Potential organoleptic effects of PLA

PLA is an organic acid and may theoretically affect the sensory profile of fish, such as odor or flavor (Supplementary Table S4). In this study, the sensory assessment focused on odor, texture, and overall acceptability; “sourness” was not separately evaluated as a taste attribute. While no off-odor was reported by panelists at the applied concentration (2.0 mg/mL), future studies should include taste panel assessments or instrumental flavor analysis (such as electronic tongue) to fully characterize any potential off-flavors associated with PLA treatment.

### 3.8 Limitations of this study

Several limitations of this study should be acknowledged. First, the sample size for non-targeted metabolomics and ML was relatively small (*n* = 6 per group), and the ratio of metabolic features to samples was high (approximately 2,847 features vs. 12 samples, p/n ≈ 237). Although nested cross-validation and external validation (*n* = 6 independent samples) were performed to mitigate overfitting, future studies with larger cohorts are needed to confirm the robustness of the identified biomarkers. Second, metabolite identification was performed at MSI level 2 (putatively annotated), and further validation using authentic standards is required. Third, the antibacterial mechanisms proposed here are based on correlative metabolomics and enzymatic assays; direct genetic evidence (such as gene knockout or complementation of key enzymes such as IDH, SDH, or MDH) is needed to establish causality. Fourth, the sensory evaluation did not include a specific assessment of sourness or off-flavors induced by PLA, and the organoleptic effects of PLA at the applied concentration should be systematically evaluated in future studies. Finally, the preservation experiments were conducted under aerobic packaging at 4 °C; the efficacy of PLA under modified atmosphere packaging or combined with other preservatives remains to be explored.

## 4 Conclusion

This study preliminarily reveals the multi-target antimicrobial mechanism of PLA against putrefactive *Shewanella* species and its potential applications in aquatic product preservation. PLA disrupts cell membrane integrity, triggering comprehensive disruption of core metabolic pathways such as the TCA cycle, amino acid metabolism, nucleotide metabolism, and cofactor synthesis. Key metabolites (*α*-ketoglutarate, NADPH, and AMP) were significantly downregulated, markedly altering the metabolic profile of *Shewanella*. All four ML models perfectly distinguished the treatment group from the control group, and SHAP analysis further confirmed that the aforementioned metabolites are key targets of PLA antibacterial activity. Application validation demonstrated that PLA treatment effectively extends the refrigerated shelf life of yellow croaker, sea bass, and salmon. Future research could further explore the synergistic effects of PLA with other natural preservatives to advance its practical application in the food industry.

## Data Availability

The original contributions presented in the study are included in the article/Supplementary material, further inquiries can be directed to the corresponding author.
