# Supplementary material for: Antibacterial mechanisms of phenyllactic acid against Shewanella putrefaciens by metabolomics and machine learning and its application in sea fish
Source: Front Nutr. 2026 Jun 29;13:1852332. doi: 10.3389/fnut.2026.1852332 (PMC13357118; doi:10.3389/fnut.2026.1852332)
Supplement: Supplementary file 2 [file Table_2.DOCX]

**Supplementary Methods S1.**

**Non-targeted metabolomics analysis method**

The non-targeted metabolomics analysis was evaluated following established protocols (1). *Shewanella putrefaciens* SP22 cells in logarithmic phase were treated with 1× MIC concentration of PLA for 24 h, then centrifuged at 4500 rpm at 4℃ for 10 min, followed by washing three times with PBS. The precipitate with bacterial cells (30±5 mg) was obtained and resuspended in300 μL of 70% methanol, ground for 6 min with a frozen tissue grinder, and submitted to cryosonication for 30 min at 5 ◦C to extract metabolites. After standing at −20 ◦C for 30 min, samples were centrifuged at 6,000 rpm at 4 ◦C for 15 min, and the supernatant was collected for UPLC-MS/MS analysis. Samples not treated with PLA were analyzed in identical manner as samples treated with PLA. Each sample was analyzed as six independent replicates. During measurements, a QC sample was inserted every eight test samples in parallel to ensure quality control of the analytical process.

Raw LC-MS/MS data was imported into ProteoWizard( https://proteowizard.sourceforge.io/) for analysis. Metabolites were identified using the Metabolism Public Database (HMDB) (http://www.hmdb.ca/) and in- house metware databases. The processed data matrix was uploaded to the metware cloud platform (https://www.metware.cn) for bio-informatic analysis. The metabolites satisfying variable importance in prediction (VIP) > 1, adjusted P < 0.05, and absolute fold change (FC) > 1 were defined as differentially accumulated metabolites (DAMs).Metabolic pathway annotation was performed using the KEGG database (http://www.genome.jp/kegg/). Pathway enrichment analyses were performed using the metware cloud platform to identify potential key biological pathways affected by PLA treatment.

1. Liang, M., H. Wang, Z. Zhou, et al., *Antibacterial mechanism of Lactiplantibacillus plantarum SHY96 cell-free supernatant against Listeria monocytogenes revealed by metabolomics and potential application on chicken breast meat preservation.* Food Chemistry: X, 2025. **25**.

**TableS2.**  Sensory attributes and their classification into main sensory modalities used for Quantitative Descriptive Analysis（QDA）

| **Main Sensory**  **Modalities** | **Sensory Attributes** |
| --- | --- |
| Appearance | color,color uniformity,paste homogeneity,presence of air bubbles,paste shininess |
| odor | Fish odor in tensity, presence of citrus notes, presence of off-odors |
| Texture | viscosity, smoothness, adhesiveness, spread ability |
| Overall acceptability | Multiple and comprehensive factors determine the overall sensory acceptability |

Note: Given that the consumer hedonic tests in this study were conducted under controlled conditions, all the steps of the hedonic sensory evaluation process adhered to the regulations of Elena-Iuliana Flocea et al. The acceptance of fish products was assessed using a 9-point hedonic scale (1 = extremely dislike, 2 = very dislike, 3 = dislike, 4 = slightly dislike, 5 = neither like nor dislike, 6 = slightly, 7 = like, 8 = very like, 9 = extremely like).

| **Table S3** Metabolite-indexed significant differential metabolites and metabolic pathways | | | | | | | | | |
| --- | --- | --- | --- | --- | --- | --- | --- | --- | --- |
| **Kegg_pathway** |  | **Index** | **Compounds** | **Formula** | **VIP** | **P-value** | **Fold_Change** | **Log2FC** | **Type** |
| **TCA cycle** | 1 | MW0106168 | Citric acid | C6H8O7 | 1.457409593 | 0.002736933 | 0.028427366 | -5.136575736 | down |
|  | 2 | MW0169880 | Succinic acid | C4H6O4 | 1.433184709 | 0.007500349 | 0.052499665 | -4.251547975 | down |
|  | 3 | MEDN0202 | α-Ketoglutaric Acid (α-KG) | C5H6O5 | 1.431706373 | 0.002397975 | 0.095293266 | -3.391481914 | down |
|  | 4 | MW0169311 | Isocitric acid | C6H8O7 | 1.471840973 | 0.002496106 | 0.016312194 | -5.937905379 | down |
|  | 5 | MW0108118 | Malic acid | C4H6O5 | 1.230730632 | 0.000733179 | 1.304751895 | 0.383775497 | up |
| **Amino acid** | 1 | MW0109196 | L-Phenylalanine | C9H11NO2 | 1.436417312 | 1.74504E-07 | 0.308873401 | -1.694912457 | down |
|  | 2 | MEDN0006 | L-Tyrosine | C9H11NO3 | 1.426071026 | 8.57797E-05 | 0.413364592 | -1.27451328 | down |
|  | 3 | MEDN1387 | O-acetyl-L-serine | C5H9NO4 | 1.45478844 | 1.64621E-05 | 0.138729278 | -2.849655807 | down |
|  | 4 | MEDP0322 | L-Homoserine | C4H9NO3 | 1.332804753 | 5.36835E-05 | 1.636595688 | 0.710697956144453 | up |
|  | 5 | MW0106168 | Citric acid | C6H8O7 | 1.457409593 | 0.002736933 | 0.028427366 | -5.136575736 | down |
|  | 6 | MW0193946 | L-Tryptophan | C11H12N2O2 | 1.479494377 | 4.82996E-07 | 0.030756239 | -5.022977091 | down |
|  | 7 | MEDN1093 | 2-Isopropylmalate | C7H12O5 | 1.461004722 | 0.000332824 | 0.053269713 | -4.230540686 | down |
|  | 8 | MW0009432 | Phenylpyruvic acid | C9H8O3 | 1.474052851 | 0.000280621 | 47.41764484 | 5.56735210313884 | up |
|  | 9 | MW0104827 | Oxoadipic acid | C6H8O5 | 1.407289255 | 4.15194E-06 | 1.192553469 | 0.254053952 | up |
|  | 10 | MW0108639 | N-Acetyl-L-glutamic acid | C7H11NO5 | 1.47553269 | 0.000208966 | 0.047253468 | -4.403435972 | down |
|  | 11 | MEDN1133 | Butanone acid | C4H6O3 | 1.299107459 | 0.020662714 | 0.060478396 | -4.047436306 | down |
|  | 12 | FDATN01488 | L-Glutamic acid | C5H9NO4 | 1.473184609 | 2.56956E-05 | 0.091172031 | -3.455264883 | down |
|  | 13 | MW0107683 | L-cystathionine | C7H14N2O4S | 1.319800076 | 0.010985196 | 2.718160091 | 1.442630429 | up |
|  | 14 | MEDN0202 | α-Ketoglutaric Acid (α-KG) | C5H6O5 | 1.431706373 | 0.002397975 | 0.095293266 | -3.391481914 | down |
|  | 15 | MW0107786 | L-Leucine | C6H13NO2 | 1.314046589 | 0.000714345 | 0.133106763 | -2.909344219 | down |
|  | 16 | MW0155528 | phosphoribosyl-AMP | C15H23N5O14P2 | 1.486946565 | 9.6735E-05 | 0.017899233 | -5.803958451 | down |
|  | 17 | MW0115233 | Ribulose 5-phosphate | C5H11O8P | 1.477064588 | 0.000642709 | 0.030633535 | -5.02874435 | down |
|  | 18 | MW0169311 | Isocitric acid | C6H8O7 | 1.471840973 | 0.002496106 | 0.016312194 | -5.937905379 | down |
|  | 19 | MW0112916 | 3-Phosphoglyceric acid | C3H7O7P | 1.448414546 | 0.00213074 | 0.074995149 | -3.737058915 | down |
| **Nucleotide metabolism** | 1 | MW0103478 | Adenosine monophosphate | C10H14N5O7P | 1.484296823 | 1.23107E-06 | 0.026554104 | -5.234921346 | down |
|  | 2 | MW0103590 | Guanosine-5'-monophosphate | C10H14N5O8P | 1.349342955 | 0.005298482 | 0.152351622 | -2.714523238 | down |
|  | 3 | MW0103709 | Uridine-5'-monophosphate | C9H13N2O9P | 1.303207257 | 0.000259186 | 0.321043932 | -1.639157364 | down |
|  | 4 | MW0103469 | Adenosine-5'-diphosphate | C10H15N5O10P2 | 1.473912215 | 1.16168E-05 | 0.123785622 | -3.014084341 | down |
|  | 5 | MW0103519 | Cytidine-5'-diphosphate | C9H15N3O11P2 | 1.448410359 | 1.41177E-06 | 0.101935786 | -3.29426747 | down |
|  | 6 | MW0124275 | Guanine | C5H5N5O | 1.172564148 | 0.003548798 | 0.125714904 | -2.991772399 | down |
|  | 7 | MW0159913 | Inosine | C10H12N4O5 | 1.250421024 | 0.012066177 | 0.394802585 | -1.340796658 | down |
|  | 8 | MW0170020 | Xanthosine | C10H12N4O6 | 1.27043705 | 4.32024E-06 | 4.078017671 | 2.027868027 | up |
|  | 9 | MW0103706 | Uridine-5'-diphosphate | C9H14N2O12P2 | 1.463652262 | 0.000164869 | 0.046059107 | -4.440369741 | down |
|  | 10 | MEDN0152 | Adenosine 5'-Diphosphate | C10H15N5O10P2 | 1.483386028 | 9.17814E-06 | 0.119866334 | -3.060501573 | down |
|  | 11 | MW0103589 | Guanosine-5'-diphosphate | C10H15N5O11P2 | 1.478559116 | 0.000170523 | 0.063065149 | -3.987013236 | down |
|  | 12 | MW0170017 | Xanthine | C5H4N4O2 | 1.15773571 | 0.00152757 | 1.828149241 | 0.87038385 | up |
|  | 13 | MW0103351 | 2'-Deoxyuridine | C9H12N2O5 | 1.462582437 | 1.41558E-06 | 1.675059082 | 0.744211983 | up |
|  | 14 | MW0114186 | Deoxycytidine diphosphate | C9H15N3O10P2 | 1.030144811 | 0.015985064 | 0.193126785 | -2.372379831 | down |
|  | 15 | pme3174 | Cytidine-5'-monophosphate | C9H14N3O8P | 1.446470217 | 8.80258E-08 | 1.805083655 | 0.852065699 | up |
|  | 16 | MEDN0602 | Deoxyguanosine 5'-monophosphate(dGMP) | C10H14N5O7P | 1.483958722 | 9.42498E-05 | 0.028915931 | -5.111991639 | down |
|  | 17 | MW0103346 | 2'-Deoxyguanosine-5'-diphosphate | C10H15N5O10P2 | 1.404064892 | 0.008907827 | 0.051590373 | -4.276754323 | down |
|  | 18 | MW0103525 | Deoxyuridine-5'-diphosphate | C9H14N2O11P2 | 1.149983378 | 0.022949976 | 0.317488438 | -1.655224043 | down |
| **Glycerophospholipid metabolism** | 1 | MEDP1002 | Acetylcholine | C7H15NO2 | 1.459453337 | 9.71865E-05 | 0.091197279 | -3.45486541 | down |
|  | 2 | MEDP1904 | LPE(18:2/0:0) | C23H44NO7P | 1.361442862 | 0.000171181 | 0.754172923 | -0.40703274 | down |
|  | 3 | MW0053733 | Glycerophospho-N-Oleoyl Ethanolamine | C23H46NO7P | 1.007803422 | 0.016455708 | 0.89426947 | -0.161218471 | down |
|  | 4 | MEDP1180 | LPC(14:0/0:0) | C22H46NO7P | 1.466025509 | 0.000343814 | 0.043274641 | -4.530334331 | down |
|  | 5 | MW0013615 | 1-octadecanoyl-2-(9Z-octadecenoyl)-sn-glycero-3-phosphoserine | C42H80NO10P | 1.164992828 | 0.00417513 | 0.845890389 | -0.241457365 | down |
|  | 6 | MEDP0638 | LPE(16:1/0:0) | C21H42NO7P | 1.480399579 | 8.43647E-05 | 0.072600224 | -3.783882199 | down |
|  | 7 | MEDL01932 | 1-Pentadecanoyl-glycero-3-phosphoethanolamine | C20H42NO7P | 1.465645875 | 0.000168971 | 0.104441799 | -3.259228886 | down |
|  | 8 | MW0058181 | 1-(9Z-heptadecenoyl)-sn-glycero-3-phosphoethanolamine | C22H44NO7P | 1.477689526 | 0.000364025 | 0.048724074 | -4.359221434 | down |
|  | 9 | MW0057009 | PC(18:1(11Z)/P-16:0) | C42H82NO7P | 1.184677212 | 0.035873073 | 0.211531059 | -2.241058585 | down |
|  | 10 | MEDN0362 | LPE(18:1/0:0) | C23H46NO7P | 1.471196902 | 0.000154225 | 0.159328882 | -2.649920286 | down |
|  | 11 | MW0054600 | LPE(16:0/0:0) | C21H44NO7P | 1.479472558 | 1.9538E-05 | 0.141252566 | -2.823651018 | down |
|  | 12 | MEDN1485 | Glycerophospho-N-Arachidonoyl Ethanolamine | C25H44NO7P | 1.320088403 | 0.003794006 | 0.493354502 | -1.019303422 | down |
|  | 13 | MW0013003 | 1-Stearoyl-2-linoleoyl-sn-glycero-3-phosphoethanolamine | C41H78NO8P | 1.035386846 | 0.019244226 | 0.457824123 | -1.127134612 | down |
|  | 14 | MW0011869 | 1,2-Dipalmitoyl-sn-glycerol 3-phosphate | C35H69O8P | 1.210857458 | 0.005642872 | 0.628189975 | -0.670727176 | down |
|  | 15 | MW0058014 | LPE(14:0/0:0) | C19H40NO7P | 1.479939047 | 0.00014283 | 0.05287844 | -4.241176585 | down |
|  | 16 | MW0013006 | 1-Stearoyl-2-myristoyl-sn-glycero-3-phosphocholine | C40H80NO8P | 1.119162168 | 0.002639476 | 0.154188333 | -2.69723449 | down |
|  | 17 | MW0012982 | LPC(15:0/0:0) | C23H48NO7P | 1.439424381 | 0.00025419 | 0.166730331 | -2.584411513 | down |
|  | 18 | MW0056784 | 1-Myristoyl-2-palmitoyl-sn-glycero-3-phosphocholine | C38H76NO8P | 1.071976382 | 0.000636968 | 0.344449601 | -1.537635188 | down |
|  | 19 | MW0012969 | 1-Palmitoyl-2-myristoyl-sn-glycero-3-phosphocholine | C38H76NO8P | 1.308977288 | 8.22401E-06 | 0.263097054 | -1.926333001 | down |
|  | 20 | MW0050667 | 1-(5Z,8Z,11Z,14Z-eicosatetraenoyl)-2-(7Z,10Z,13Z,16Z-docosatetraenoyl)-sn-glycerol | C45H72O5 | 1.153523496 | 0.012361466 | 0.568354368 | -0.815137366 | down |
|  | 21 | MEDP1339 | LPC(18:1/0:0) | C26H52NO7P | 1.158549392 | 0.013105351 | 1.444408425 | 0.53047874 | up |
|  | 22 | MEDP1122 | MG(16:0/0:0/0:0) | C19H38O4 | 1.243134031 | 0.000385275 | 1.709681222 | 0.77372735292433 | up |
|  | 23 | MW0013543 | 1-hexadecanoyl-2-(9Z,12Z-octadecadienoyl)-sn-glycero-3-phosphoethanolamine | C39H74NO8P | 1.287292514 | 0.001447632 | 9.15208651 | 3.194100689 | up |
|  | 24 | MW0013612 | 1-Palmitoyl-2-glutaryl-sn-glycero-3-phosphocholine | C29H56NO10P | 1.298390377 | 0.001563791 | 1.680717663 | 0.749077392 | up |
|  | 25 | MEDP0125 | Choline | C5H14NO+ | 1.260258967 | 0.004685262 | 1.183605107 | 0.243187826 | up |
|  | 26 | MW0012966 | 1-Palmitoyl-2-linoleoyl-sn-glycero-3-phosphate | C37H69O8P | 1.136236544 | 0.017042178 | 2.764781783 | 1.467165617 | up |
|  | 27 | MW0056906 | 1-Palmitoyl-2-docosahexaenoyl-sn-glycero-3-phosphocholine | C46H80NO8P | 1.036900499 | 0.015159738 | 1.771479894 | 0.824955091 | up |
|  | 28 | MW0012973 | 1-Palmitoyl-2-oleoyl-sn-glycero-3-phosphate | C37H71O8P | 1.284728735 | 0.000241195 | 1.292108058 | 0.369726726 | up |
|  | 29 | MW0050069 | 1-Vaccenoyl-2-pentadecanoyl-sn-glycerol | C36H68O5 | 1.171374752 | 0.002189525 | 1.629048747 | 0.704029774857695 | up |
| **Biosynthesis of cofactors** | 1 | MW0103478 | Adenosine monophosphate | C10H14N5O7P | 1.484296823 | 1.23107E-06 | 0.026554104 | -5.234921346 | down |
|  | 2 | MW0126334 | Pyridoxal phosphate | C8H10NO6P | 1.452825689 | 7.79433E-06 | 0.283593011 | -1.818106115 | down |
|  | 3 | MEDN0006 | L-Tyrosine | C9H11NO3 | 1.426071026 | 8.57797E-05 | 0.413364592 | -1.27451328 | down |
|  | 4 | MW0103709 | Uridine-5'-monophosphate | C9H13N2O9P | 1.303207257 | 0.000259186 | 0.321043932 | -1.639157364 | down |
|  | 5 | MW0103469 | Adenosine-5'-diphosphate | C10H15N5O10P2 | 1.473912215 | 1.16168E-05 | 0.123785622 | -3.014084341 | down |
|  | 6 | MW0159685 | Glutathione | C10H17N3O6S | 1.45930959 | 6.70457E-05 | 0.070737983 | -3.821371104 | down |
|  | 7 | MW0103519 | Cytidine-5'-diphosphate | C9H15N3O11P2 | 1.448410359 | 1.41177E-06 | 0.101935786 | -3.29426747 | down |
|  | 8 | MEDP1010 | NADP+ | C21H28N7O17P3 | 1.467074494 | 6.48671E-05 | 0.136936099 | -2.868425271 | down |
|  | 9 | MW0103557 | Flavin adenine dinucleotide | C27H33N9O15P2 | 1.477065618 | 4.51123E-07 | 0.040368712 | -4.630618628 | down |
|  | 10 | MW0109026 | Oxiglutatione | C20H32N6O12S2 | 1.480515218 | 0.000326839 | 0.044947482 | -4.475615905 | down |
|  | 11 | MW0103635 | Nicotinic acid adenine dinucleotide | C21H27N6O15P2+ | 1.476458071 | 3.13646E-06 | 0.036783631 | -4.764792295 | down |
|  | 12 | MW0005091 | 4-Hydroxybenzoic acid | C7H6O3 | 1.438441619 | 1.73382E-07 | 0.334473825 | -1.58003478 | down |
|  | 13 | MW0106168 | Citric acid | C6H8O7 | 1.457409593 | 0.002736933 | 0.028427366 | -5.136575736 | down |
|  | 14 | MW0103561 | Flavin mononucleotide | C17H21N4O9P | 1.36044079 | 3.09882E-05 | 0.047086838 | -4.408532334 | down |
|  | 15 | MW0193946 | L-Tryptophan | C11H12N2O2 | 1.479494377 | 4.82996E-07 | 0.030756239 | -5.022977091 | down |
|  | 16 | MW0103706 | Uridine-5'-diphosphate | C9H14N2O12P2 | 1.463652262 | 0.000164869 | 0.046059107 | -4.440369741 | down |
|  | 17 | MEDN0152 | Adenosine 5'-Diphosphate | C10H15N5O10P2 | 1.483386028 | 9.17814E-06 | 0.119866334 | -3.060501573 | down |
|  | 18 | MADN0568 | Uridine-5'-diphosphoglucuronic Acid (sodium salt hydrate) | C15H22N2O18P2 | 1.475436411 | 0.00046842 | 0.032996542 | -4.921541347 | down |
|  | 19 | MW0104827 | Oxoadipic acid | C6H8O5 | 1.407289255 | 4.15194E-06 | 1.192553469 | 0.254053952 | up |
|  | 20 | MW0126398 | Quinolinic acid | C7H5NO4 | 1.439273278 | 0.007135594 | 0.065385819 | -3.93487841 | down |
|  | 21 | FDATN01488 | L-Glutamic acid | C5H9NO4 | 1.473184609 | 2.56956E-05 | 0.091172031 | -3.455264883 | down |
|  | 22 | MW0107683 | L-cystathionine | C7H14N2O4S | 1.319800076 | 0.010985196 | 2.718160091 | 1.442630429 | up |
|  | 23 | MW0124668 | L-Gulonolactone | C6H10O6 | 1.035967238 | 0.040881436 | 1.474743195 | 0.560463752 | up |
|  | 24 | MEDN0202 | α-Ketoglutaric Acid (α-KG) | C5H6O5 | 1.431706373 | 0.002397975 | 0.095293266 | -3.391481914 | down |
|  | 25 | MW0007325 | Homogentisic acid | C8H8O4 | 1.267453029 | 0.000465489 | 1.460041781 | 0.546009654 | up |
|  | 26 | MW0114745 | L-Kynurenine | C10H12N2O3 | 1.181818193 | 0.000613689 | 0.537042897 | -0.896890766 | down |
|  | 27 | MW0004631 | p-Aminobenzoic acid | C7H7NO2 | 1.474377335 | 0.001247305 | 0.036855682 | -4.761969125 | down |
|  | 28 | MW0103684 | Uridine-5'-diphosphate-glucose | C15H24N2O17P2 | 1.46919907 | 0.000736073 | 0.068206817 | -3.873940245 | down |
|  | 29 | MW0115233 | Ribulose 5-phosphate | C5H11O8P | 1.477064588 | 0.000642709 | 0.030633535 | -5.02874435 | down |
|  | 30 | MW0169311 | Isocitric acid | C6H8O7 | 1.471840973 | 0.002496106 | 0.016312194 | -5.937905379 | down |
|  | 31 | MW0112916 | 3-Phosphoglyceric acid | C3H7O7P | 1.448414546 | 0.00213074 | 0.074995149 | -3.737058915 | down |
|  | 32 | MW0143562 | 5-amino-6-(5-phospho-D-ribitylamino)uracil | C9H17N4O9P | 1.190268545 | 0.025293182 | 0.426532473 | -1.229272513 | down |
|  | 33 | MW0103543 | Dihydroflavine-adenine dinucleotide | C27H35N9O15P2 | 1.407525818 | 4.95871E-07 | 0.069401976 | -3.848879455 | down |
|  | 34 | MW0143561 | 5-amino-6-(D-ribitylamino)uracil | C9H16N4O6 | 1.030326179 | 0.010723699 | 1.629932021 | 0.704811796 | up |
|  | 35 | MW0103633 | Nicotinate mononucleotide | C11H15NO9P+ | 1.165559609 | 0.004662879 | 12.79276406 | 3.677256108 | up |
|  | 36 | MW0103583 | Guanosine diphosphate mannose | C16H25N5O16P2 | 1.312320554 | 0.011582231 | 0.134868493 | -2.890374743 | down |
|  | 37 | MW0143251 | (4-Amino-2-methylpyrimidin-5-yl)methyl trihydrogen diphosphate | C6H11N3O7P2 | 1.383936145 | 0.000123375 | 1.25911733 | 0.332412725 | up |
|  | 38 | MW0161649 | (1R,10R,12S,17R)-5-amino-11,11,14-trihydroxy-14-oxo-13,15,18-trioxa-2,4,6,9-tetraza-14lambda5-phosphatetracyclo[8.8.0.03,8.012,17]octadeca-3(8),5-dien-7-one | C10H14N5O8P | 1.442171541 | 0.000757729 | 0.130090353 | -2.942414116 | down |
|  | 39 | MW0103308 | 1-Deoxy-1-(7,8-dimethyl-2,4-dioxo-3,4-dihydro-2H-benzo[G]pteridin-1-ID-10(5H)-YL)-5-O-phosphonato-D-ribitol | C17H23N4O9P | 1.481597688 | 0.000273869 | 0.057680127 | -4.115781843 | down |
|  | 40 | MW0103628 | NADP nicotinamide-adenine-dinucleotide phosphate | C21H29N7O17P3+ | 1.478916303 | 0.00029557 | 0.033887679 | -4.883095353 | down |
|  | 41 | MW0158760 | UDPgalacturonate | C15H22N2O18P2 | 1.477114829 | 0.000173907 | 0.057645261 | -4.116654177 | down |
|  | 42 | MW0103901 | (3R)-3-Hydroxy-2-oxo-4-phosphonooxybutanoate | C4H7O8P | 1.237214312 | 0.002699837 | 2.342015868 | 1.227750851 | up |
|  | 43 | MW0143563 | 5-amino-6-(5-phospho-beta-D-ribosylamino)uracil | C9H15N4O9P | 1.403737622 | 0.001161174 | 8.446483996 | 3.078350918 | up |
| **Teichoic acid biosynthesis** | 1 | MEDP1122 | MG(16:0/0:0/0:0) | C19H38O4 | 1.243134031 | 0.000385275 | 1.709681222 | 0.773727353 | up |
|  | 2 | MEDP0125 | Choline | C5H14NO+ | 1.260258967 | 0.004685262 | 1.183605107 | 0.243187826 | up |
|  | 3 | MW0050069 | 1-Vaccenoyl-2-pentadecanoyl-sn-glycerol | C36H68O5 | 1.171374752 | 0.002189525 | 1.629048747 | 0.704029775 | up |
|  | 4 | MW0050667 | 1-(5Z,8Z,11Z,14Z-eicosatetraenoyl)-2-(7Z,10Z,13Z,16Z-docosatetraenoyl)-sn-glycerol | C45H72O5 | 1.153523496 | 0.012361466 | 0.568354368 | -0.815137366 | down |
| **Pyruvate metabolism** | 1 | MW0169880 | Succinic acid | C4H6O4 | 1.433184709 | 0.007500349 | 0.052499665 | -4.251547975 | down |
|  | 2 | MEDN1093 | 2-Isopropylmalate | C7H12O5 | 1.461004722 | 0.000332824 | 0.053269713 | -4.230540686 | down |
|  | 3 | MW0108118 | Malic acid | C4H6O5 | 1.230730632 | 0.000733179 | 1.304751895 | 0.383775497 | up |
|  | 4 | MW0105420 | Acetylenedicarboxylic acid | C4H2O4 | 1.323866817 | 5.61822E-05 | 0.551370512 | -0.858905984 | down |
| **Fatty acid degradation** | 1 | MW0169232 | Glutaric acid | C5H8O4 | 1.422207514 | 2.45353E-07 | 0.27453319 | -1.864947519 | down |
|  | 2 | MW0054414 | L-Palmitoylcarnitine | C23H45NO4 | 1.070384816 | 0.000442228 | 2.268830943 | 1.181949114 | up |
|  | 3 | MW0053909 | Hexanoyl-coenzyme a | C27H46N7O17P3S | 1.481030542 | 0.00064707 | 0.046822601 | -4.416651123 | down |
|  | 4 | MEDN0381 | FFA(16:0) | C16H32O2 | 1.308149054 | 0.000917203 | 1.647998157 | 0.72071463 | up |
| **Fatty acid biosynthesis** | 1 | MW0054288 | Dodecanoic acid | C12H24O2 | 1.115883806 | 0.001577092 | 1.822331903 | 0.865785742 | up |
|  | 2 | MEDP0904 | FFA(18:1) | C18H34O2 | 1.103529244 | 0.020841931 | 1.717487295 | 0.780299427 | up |
|  | 3 | MEDN0381 | FFA(16:0) | C16H32O2 | 1.308149054 | 0.000917203 | 1.647998157 | 0.72071463 | up |
| **Fatty acid metabolism** |  |  |  |  |  |  |  |  |  |
|  | 1 | MW0054414 | L-Palmitoylcarnitine | C23H45NO4 | 1.070384816 | 0.000442228 | 2.268830943 | 1.181949114 | up |
|  | 2 | MEDP0904 | FFA(18:1) | C18H34O2 | 1.103529244 | 0.020841931 | 1.717487295 | 0.780299427 | up |
|  | 3 | MEDN0381 | FFA(16:0) | C16H32O2 | 1.308149054 | 0.000917203 | 1.647998157 | 0.72071463 | up |
| **Fatty acid elongation** | 1 | MW0103463 | Adenosine-3'-5'-diphosphate | C10H15N5O10P2 | 1.298869647 | 0.004307776 | 0.082273934 | -3.603420757 | down |
|  | 2 | MEDN0381 | FFA(16:0) | C16H32O2 | 1.308149054 | 0.000917203 | 1.647998157 | 0.72071463 | up |
| **Pantothenate and CoA biosynthesis** |  |  |  |  |  |  |  |  |  |
|  | 1 | MW0103463 | Adenosine-3'-5'-diphosphate | C10H15N5O10P2 | 1.298869647 | 0.004307776 | 0.082273934 | -3.603420757 | down |
| **Biofilm formation - Pseudomonas aeruginosa** | 1 | MADP0537 | cyclic di-GMP | C20H24N10O14P2 | 1.299313923 | 1.90994E-06 | 7.450083451 | 2.897256586 | up |
|  | 2 | MW0103508 | Cyclic AMP | C10H12N5O6P | 1.452522387 | 0.0071932 | 0.020925998 | -5.57855974 | down |

**TableS4** Mean sensory scores (±SD) of fishes samples and significance of samples,sensory attribute,and their interaction.

| Fish species | | Storage time (days) | Appearance | Odor | Texture | Overall acceptability |
| --- | --- | --- | --- | --- | --- | --- |
| Large yellow croaker | CK | 0 | 8.76±0.08 a | 8.73±0.12 a | 8.78±0.17 a | 8.74±0.21 a |
|  |  | 2 | 8.13±0.11 b | 7.72±0.11 b | 7.79±0.30 b | 7.31±0.17 c |
|  |  | 4 | 6.07±0.31 d | 5.74±0.12 e | 5.81±0.10 e | 5.57±0.11 f |
|  |  | 6 | 2.45±0.22 e | 3.78±0.21 g | 2.79±0.12 g | 3.46±0.07 g |
|  |  | 8 | 1.29±0.35 f | 0.91±0.12 h | 1.06±0.09 h | 1.73±0.28 h |
|  | PLA（MIC） | 0 | 8.67±0.07 a | 8.69±0.13 a | 8.71±0.02 a | 8.80±0.03 a |
|  |  | 2 | 8.32±0.14 b | 7.82±0.22 b | 7.07±0.17 c | 8.02±0.09 b |
|  |  | 4 | 7.52±0.16 c | 7.36±0.08 c | 6.55±0.16 d | 7.32±0.17 c |
|  |  | 6 | 7.23±0.19 c | 6.94±0.11 d | 6.01±0.15 e | 6.53±0.11 d |
|  |  | 8 | 6.25±0.26 d | 4.78±0.02 f | 4.90±0.19 f | 6.00±0.06 e |
| Salmon | CK | 0 | 8.40±0.25 a | 8.69±0.25 a | 8.71±0.12 a | 8.70±0.11 a |
|  |  | 2 | 6.23±0.23 e | 5.82±0.23 d | 5.07±0.15 e | 6.27±0.16 d |
|  |  | 4 | 5.12±0.14 f | 3.60±0.19 f | 3.92±0.09 f | 4.69±0.18 e |
|  |  | 6 | 1.97±0.14 g | 1.60±0.17 g | 1.92±0.08 g | 1.33±0.40 f |
|  |  | 8 | 1.30±0.34 h | 1.02±0.02 h | 1.23±0.07 h | 1.02±0.02 f |
|  | PLA（MIC） | 0 | 8.67±0.11 a | 8.77±0.28 a | 8.79±0.19 a | 8.84±0.05 a |
|  |  | 2 | 8.06±0.05 b | 7.36±0.08 b | 7.55±0.16 b | 8.10±0.07 b |
|  |  | 4 | 7.56±0.11 c | 6.92±0.09 c | 7.16±0.05 c | 7.59±0.01 c |
|  |  | 6 | 7.08±0.08 d | 5.90±0.10 d | 6.09±0.18 d | 6.38±0.18 d |
|  |  | 8 | 6.15±0.19 e | 4.77±0.17 e | 4.85±0.12 e | 4.75±0.13 e |
| Sea Bass | CK | 0 | 8.80±0.05 a | 8.76±0.20 a | 8.83±0.31 a | 8.74±0.09 a |
|  |  | 2 | 6.64±0.07 e | 6.94±0.11 c | 7.01±0.15 c | 6.57±0.11 d |
|  |  | 4 | 5.23±0.09 g | 4.12±0.13 f | 4.35±0.22 f | 4.93±0.12 f |
|  |  | 6 | 2.32±0.16 h | 2.23±0.09 g | 2.09±0.11 g | 2.77±0.07 h |
|  |  | 8 | 1.12±0.04 i | 1.32±0.02 h | 1.54±0.09 h | 1.57±0.01 i |
|  | PLA（MIC） | 0 | 8.63±0.14 a | 8.81±0.20 a | 8.72±0.31 a | 8.73±0.09 a |
|  |  | 2 | 8.08±0.04 b | 7.93±0.22 b | 7.52±0.19 b | 7.69±0.05 b |
|  |  | 4 | 7.62±0.09 c | 6.62±0.12 d | 6.51±0.14 d | 7.12±0.11 c |
|  |  | 6 | 6.30±0.19 a | 6.91±0.07 b | 6.06±0.11 b | 6.29±0.07 b |
|  |  | 8 | 5.76±0.09 a | 4.41±0.03 b | 4.36±0.21 b | 3.56±0.12 a |

Different letters represent different levels of significance. *P*<0.05.
